# Neutrophils in traumatic brain injury (TBI): friend or foe?

**DOI:** 10.1186/s12974-018-1173-x

**Published:** 2018-05-17

**Authors:** Yang-Wuyue Liu, Song Li, Shuang-Shuang Dai

**Affiliations:** 1 Department of Biochemistry and Molecular Biology, Army Medical University, Chongqing, 400038 People’s Republic of China; 2 Center for Pharmacogenetics, School of Pharmacy, University of Pittsburgh, Pittsburgh, Pennsylvania 15261 USA; 3 Molecular Biology Center, State Key Laboratory of Trauma, Burn, and Combined Injury, Daping Hospital, Army Medical University, Chongqing, 400042 People’s Republic of China

**Keywords:** Neutrophils, Polymorphonuclear cell, Traumatic brain injury, Edema, Neuroinflammation, Blood-brain barrier, Neurodegeneration, Nerve recovery

## Abstract

Our knowledge of the pathophysiology about traumatic brain injury (TBI) is still limited. Neutrophils, as the most abundant leukocytes in circulation and the first-line transmigrated immune cells at the sites of injury, are highly involved in the initiation, development, and recovery of TBI. Nonetheless, our understanding about neutrophils in TBI is obsolete, and mounting evidences from recent studies have challenged the conventional views. This review summarizes what is known about the relationships between neutrophils and pathophysiology of TBI. In addition, discussions are made on the complex roles as well as the controversial views of neutrophils in TBI.

## Background

Traumatic brain injury (TBI) is a complex injury that causes the marked brain pathology, disruption of the normal function of the brain, and death. It is an important public health issue considering not only its high mortality rate but also the numerous complications in the patients who survive the injury [1, 2], such as hospital-acquired infection, injury-related dementia, hemiplegia, and depression. These complications lead to the economic burden of society and impact the live quality of the individuals and their families [3]. Several risk factors are associated with the development and recovery of TBI, including the age, race, and gender, most of them are unchangeable factors [2]. Even though the risk factors and primary injury cannot be controlled, secondary brain damage can be reduced. Secondary injury after primary insult is an important therapeutic window that determines the development and recovery of TBI. The alteration of immune system post-TBI has been shown to play important roles in the initiation and development of secondary injury after TBI [1, 4]. Neutrophils, as the major component of the innate immune system, are regarded as the short-lived players in acute inflammation that fight against the pathogens and cause indiscriminative damage to the tissue. However, mounting evidence indicates that neutrophils are non-negligible cells that connect the innate and adaptive immune systems, promote tissue recovery, and play critical roles in anti-inflammation and chronic inflammation responses [5, 6]. In addition, neutrophils can either contribute to repair mechanisms or exacerbate the pathophysiology of trauma dependent on the stage of injury [7]. In this review, we will summarize what is known about the function of neutrophils in CNS with a focus on the discussion of their pivotal roles in the pathogenesis of TBI.

### Neutrophils: origin and physiological functions in the healthy brain

Neutrophils are the most abundant circulating granulocytes in mammals. Neutrophils, basophils, and eosinophils are also called polymorphonuclear cells (PMNs) [8]. The PMNs are generated and matured in the bone marrow, involving multiple stages including myeloid precursors, promyelocyte, myelocyte, metamyelocyte, band cell, and finally, polymorphonuclear cells [9]. Physiologically, mature neutrophils are located in the bone marrow, spleen, liver, and lung, where they are also cleared up from the circulation. In the central nervous system (CNS), neutrophils are rarely found in the brain parenchyma due to the existence of blood-brain barrier (BBB) [6]. In some specific compartments like cerebrospinal fluid (CSF), meninges and pia membrane, there are a small number of neutrophils and other immune cells that provide the immune surveillance. However, under pathological conditions like infections, trauma, ischemia, and hemorrhage, increased numbers of neutrophils enter into brain tissue [10].

The differentiation and maturation of neutrophils are tightly connected with neutrophils’ functions. They can be modulated by various endogenous factors, such as granulocyte-colony stimulating factor (G-CSF) and granulocyte-macrophage-colony stimulating factor (GM-CSF) [11], or external stimuli including stress, trauma, drugs and radiation [12, 13]. With the differentiation and maturation, there are changes in the expression levels of various membrane proteins, which are important for neutrophils to sense the danger or infection signals, and transmigrate to the targeted tissues, as well as phagocytose tissue debris. For instance, in matured and activated neutrophils, integrin β1, as well as C-X-C chemokine receptor 4 (CXCR4), is downregulated, while CXCR2, Toll-like receptor 4, *N*-formyl-leucyl-phenylalanine receptor, CD11b, and CD35 are upregulated [14, 15]. Besides the membrane receptors, various granule proteins undergo significant changes in their expression levels, including matrix metalloproteinases (MMPs), myeloperoxidase, neutrophils elastase (NE), neutrophils gelatinase-associated lipocalin, and SGP28. These are important degranulated molecules for neutrophils to fight against pathogens [14, 16, 17]. In addition to the degranulation and phagocytosis, neutrophils extracellular trap (NET) is another defensive mechanism for neutrophils to eliminate pathogens, which is composed of the histones, enzymes, and granules. The accumulated neutrophils eliminate pathogens through phagocytosis, degranulation, and NETs at target tissues, forming the first line defender of the innate immune system. Actually, neutrophils themselves could amplify their activation in an autocrine dependent manner (platelet-activating factor, leukotriene B4 and IL-18), rendering it difficult to stop immediately after the danger signal has already disappeared [18–20]. This process would lead to tissue damage indiscriminately by neutrophils. So the neutrophils are often phagocytosed or inhibited by macrophages or lymphocytes after digestion of pathogens to minimize the tissue damage.

The neutrophil-derived cytokines are quite complicated and vary depending on the underlying stimuli and tissues involved [6]. For example, brain trauma is associated with a specific alteration of surface phenotype, chemotaxis, and phagocytosis, which is quite different with that of a typical inflammation [4, 21, 22]. In addition to the common inflammation-related cytokines (Tumor necrosis factor (TNF) family; pro-inflammatory cytokines; CXC- chemokines; CC- chemokines), other anti-inflammatory cytokines, immunoregulatory cytokines, angiogenic, and neurotrophic factors are detected in neutrophils as well [23–29]. Table 1 summarizes the neutrophil-derived chemokines and molecules and their functions in brain injury. Recently, neutrophils are also regarded as an important player in cancer, autoimmune diseases, and chronic inflammation, challenging our traditional views about neutrophils [6, 30]. Thus, neutrophils need to be re-evaluated for their roles in several diseases, including TBI.

**Table 1:** Summary of the neutrophil-derived mediators and their functions in brain injury

Name	Effects	References
IL-1α	^#^IL-1α expression is closely associated with areas of BBB breakdown and neuronal death at stage of injury ^&^Induces angiogenesis in brain endothelial cells	[182, 212]
IL-1β	^#^Localizes with the area of neurons loss and might induce neurons death directly ^<^Induces astrocytes to secret hemolymphopoietic cytokines (IL-6, IL-8)	[23, 77, 212]
IL-3	^&^Suppresses secondary degeneration caused by TBI	[213]
IL-4	^&^Induces M2-polarization of microglia or macrophages ^&^Promotes white matter integrity and long-term neurological recovery	[26, 214]
IL-6	^&^Triggers nerve growth factor production in astrocytes ^<^Might be a prognosis marker of TBI patient	[66, 215]
IL-7	^<^Contributes to injury-induced reactive gliosis	[216]
IL-9	^#^Exacerbates excitotoxic brain damage	[217]
IL-10	^&^IL-10 in traumatic brain and CSF both increases significantly during the first days and may downregulate pro-inflammatory cytokines following traumatic brain damage.	[25]
IL-12	^#^Highly increased in nonsurvival TBI patients	[215]
IL-16	^<^Activates microglia and lymphocytes ^<^Promotes activated microglia and lymphocytes accumulated in microvessels	[218]
IL-17	^<^Promotes neutrophils invasion	[219]
IL-18	^<^Peaks at 7 to 14 days after injury and might participate in delayed neuroinflammation ^#^Induces brain injury in caspase-1-dependent manner ^<^Induces neutrophils themselves to secrete inflammatory related cytokines	[18, 220, 221]
IL-23	^#^Leads to brain damage and neurological deficits	[222]
CXCL1	^<^Recruits circulating-neutrophils into injured brain	[24, 61, 223]
CXCL2	^<^Peaks at 4 h after TBI, chemotactic for polymorph nuclear leukocytes	[61, 224]
CXCL3	^<^Promotes neutrophils to migrate across epithelial barriers	[61]
CXCL4	^<^Induces macrophage to differentiate into a unique phenotype	[225, 226]
CXCL5	^#^Increases microglia activation as well as BBB damage ^#^Jeopardizes myelination and promotes astrogliosis	[227]
CXCL8	^<^Promotes neutrophils to infiltrate into brain parenchyma	[228]
CXCL9	^<^Promotes lymphocytes to collaborate with mesenchymal stem cells to inhibit T cells’ functions	[229]
CXCL10	^<^Promotes blood-derived monocytes to accumulate around perivascular vessels	[230, 231]
CXCL11	^&^Promotes regenerative processes	[230]
CCL2	^<^Promotes macrophage to infiltrate into parenchyma ^<^Peaks at 8–12 h after TBI ^<^Induces transmigration of monocytes and macrophages across BBB ^<^Activates and induces chemotaxis of T cells and monocytes	[224, 228, 230, 232]
CCL3	^<^Peaks at 4 h after injury ^<^Activates and induces chemotaxis of T cells and monocytes ^<^Recruits CCR2-positive leukocytes to injured brain	[232, 233]
CCL4	^<^Activates and induces chemotaxis of T cells and monocytes	[232]
CCL17	^<^Participates in leukocytes recruitment	[234]
CCL22	^<^Participates in leukocytes recruitment	[234]
G-CSF	^&^Anti-inflammatory ^<^Promotes myeloid differentiation and M-CSF secretion ^<^Reduces T cells’ infiltration ^&^Prolongs neuronal survival	[175, 235, 236]
M-CSF	^#^Promotes microglia activation	[236, 237]
GM-CSF	^&^Suppresses secondary degeneration caused by TBI	[213]
HGF	^&^Promotes survival reconstruction of specific neurons in response to cerebral injury	[176]
TGF-α	^&^Induces proliferation, migration, and differentiation of neural stem cells after neurons damage	[238]
TGF-β	^&^Down-modulates cellular activation ^&^Blocks inflammatory responses ^&^Plays a role in nerve regeneration by stimulating nerve growth factor production as well as controlling the astrocytosis and scar formation after injury	[25, 173, 186]
VEGF	^&^Promotes angiogenesis as well as brain plasticity ^&^Increases the expression of BDNF in brain endothelial cells ^#^Enhance the leakage of BBB	[27, 180, 185]
Prokineticin 2	^&^Participates in constitutive and injury-induced neurogenesis ^#^Might promote over-inflammation	[239, 240]
TNF-α	^#^Induces astrocytes to secret hemolymphopoietic cytokines (IL-6, IL-8) ^#^Mediates PMN-driven neurotoxicity directly ^<^Early pro-apoptotic effect in neutrophils	[23, 241, 242]
Arginase	^&^Anti-inflammation ^&^Augments neurite growth	[26]
BDNF	^&^Increases cell proliferation ^&^Upregulates expression of growth factors and induces neurogenesis	[27, 243, 244]
Midkine	^&^Inhibits apoptosis of neurons ^&^Promotes neurite extension	[179, 245]
Oncostatin M	^#^Induces the expression of IL-6 and MMPs ^&^Protects neurons from excitotoxic injury	[246, 247]
NGF	^&^Supports neurons survival and nerve growth	[25, 177]
NT4	^&^Prevents neuronal cell death after TBI	[28, 178]
ROS	^#^Enhances BBB dysfunction ^#^Participates in brain energy perturbation (glucose, lactate glycerol) ^#^Leads to neurons cell death ^#^Induces microglia activation	[97, 119, 131, 241, 248]
iNOS	^<^Leads to vasodilatation of blood vessel and improve microcirculation after TBI ^&^Works as an endogenous antioxidant ^<^Contributes to protein nitrosylation and nitration	[249, 250]
MMP9	^#^Breaks down the integrity of BBB ^<^Promotes infiltration of neutrophils ^&^Participates in vessel remodeling and angiogenesis ^#^Mediates PMN-driven neurotoxicity directly	[57, 165, 184, 241, 251]
MPO	^<^Reflects the infiltration of neutrophils in brain tissue	[189]
Cathepsins	^#^Contributes to TBI-induced cell death through the programmed cell necrosis and mitochondria-mediated apoptotic pathways	[252]
Defensins	^#^Penetrates a considerable distance to disrupt the BBB sites	[120]
Cathelicidin	^<^Attracts peripheral blood neutrophils, monocytes, and T cells ^<^Promotes IL-1β processing and release	[121, 253]
NE	^#^Causes cellular stress (astrocytes and microglia) in the injured brain ^#^Induces acute neurons death	[111, 166, 254]

^#^Detrimental to the brain tissue and recovery

^&^Beneficial to the brain tissue and recovery

^<^Neutral or hard to judge

### Traumatic brain injury (TBI)

TBI is an uncontrollable event that alters the brain structure and functions dramatically. It is caused by external forces like mechanical forces and acceleration or deceleration forces [31]. The brain is an immune-privileged organ under normal condition due to following three reasons: the existence of BBB, the lack of obvious lymphatic vessels, and limited numbers of antigen-presenting cells [10]. However, trauma could cause a direct damage to BBB, resulting in infusion of a large number of peripheral antigen-presenting cells, as well as instant activation of microglia in situ [2, 32]. Recent research shows the existence of lymphatic vasculature in meninges in the central nervous system, suggesting another new route for peripheral immune cells to enter and exit brain [33]. All of these disrupt the well-balanced immune-privileged environment of CNS, forcing the communication between CNS and peripheral immune system under TBI [1].

TBI is also a complicated pathological process that is caused by primary and secondary brain damage focally or diffusely [2, 31, 32]. The primary damage of TBI is the result of the kinetic forces on the brain tissue, leading to the deformations of axons, vessels, and brain cells. The interrupted axons would trigger swelling by accumulating transported materials at the moment of injury [34]. The destroyed blood vessels pour out the blood content indiscriminately, impairing the blood supply and integrity of BBB. The damaged neuronal and glial cells could release various inflammatory factors and neurotransmitters to induce a cascade inflammatory response. All the processes in primary damage are tightly associated with the secondary damage, including alteration in blood flow (hemorrhage, ischemia), continuous breakdown of BBB, dysregulation of CSF, dysfunction of brain tissue metabolism (hypoxia, edema), neuroinflammation, and cell damage (excitotoxicity, oxidative stress, free radical production, neuronal apoptosis/necrosis) (Table 2).

**Table 2:** Therapeutic approaches of neutrophil-dependent processes in brain injury

Neutrophil’s processes	Approaches	Protective	References
Maturation differentiation and elimination	Ly6G/Gr-1 antibody	Not clear	[113, 255]
	G-CSF	Not clear	[193, 256]
	Progesteron	Yes	[257]
Activation	TLR antagonist	Yes	[196, 258]
	P-selectin blockage	Yes	[82]
	VLA-4 blockage	Yes	[259–261]
	IL-1 receptor inhibitor	Not clear	[262–264]
	IL-8 receptor inhibitor	Yes	[265]
	TNF inhibitor	Yes	[266, 267]
	IL-10	Yes	[268]
Migration	C5a or C3 antagonist	Yes	[269, 270]
	CXCR2 or CXCR4 inhibitor	Not clear	[85, 86, 271]
	TGF-β1	Yes	[272]
	Mac-1 antibody	Yes	[81, 273]
	ICAM-1 antibody	Yes	[79, 80]
Cell killing	MMP inhibitor	Yes	[93, 94]
	ROS inhibitor	Yes	[98]
	NO inhibitor	Not clear	[99, 249, 250, 274]
	NE inhibitor	Yes	[111, 166, 254]

## Roles of neutrophils in TBI

### Neutrophils and blood flow

Previous studies in animals and humans indicated that focal or global cerebral hypoperfusion frequently occurred at the early stage of TBI [35, 36]. Cerebral hypoperfusion is also associated with poor neurological outcome after severe TBI [37, 38]. The hypoperfusion of blood flow enhances the interactions of neutrophils with blood vessels [39] and promotes neutrophils to tumble and adhere by inducing the expression of L-selectin and intercellular adhesion molecule 1 (ICAM-1) in endothelial cells [40, 41]. Neutrophils influence microcirculation rheology at sites where vessel diameters are around 4–100 μm. To some extent, the quiescent and activated neutrophils are important to sustain pressure of micro vessels [42]. This may partially explain why the neutropenic rats exhibit reduced blood flow pressure in injured hemisphere compared with normal rats within 24 h after TBI [43]. Furthermore, the activated neutrophils could form pseudopods and bind to endothelium and platelets, hindering blood flow through the microvasculature [44]. This will lead to a vicious cycle of hypoperfusion and neutrophils adherence, promoting the development of ischemia or early coagulopathy [45, 46]. Palmer et al. pointed out that neutrophils contribute to vascular dysfunction either during the insult or early hours (< 4–8 h) instead of 24 h in the hypoxia-ischemia model [47]. The exact time frame during which neutrophils participate in hypoperfusion needs to be further investigated.

Hyperemia/hyperperfusion (cerebral blood flow (CBF) > 55 ml/100 g/min) may occur immediately in some animal models of moderate TBI [2, 38, 48], which could convert to ischemia subsequently. While some study indicated that the mean CBF of patients could increase from 24 h to 6 days after TBI as well [38, 49]. It seems that phasic elevation in CBF after injury is a protective mechanism for achieving functional recovery [49], and elevated CBF does not cause an increase in intracranial pressure if the auto-regulatory mechanism in the brain is well retained [50], and elevated CBF does not cause an increase in intracranial pressure if the auto-regulatory mechanism in the brain is well retained [50]. The point is that hyperemia is tightly associated with the peak rate of neutrophils influx and the degree of hyper permeability [51, 52], which is undeniably involved in enhanced neutrophils infiltration, cerebral edema, or intracerebral hemorrhage in acute phase [42, 53]. Of note, reoccurrence of hemorrhage could localize at the initial injury lesion, expand or even develop new non-contiguous hemorrhagic lesions, which make it the most life-threatening complication in TBI patients [54]. Some studies showed that neutrophils are strongly associated with hemorrhagic areas with local high activities of MMP-9 as well as degradation of basal lamina collagen IV [55–57], which deteriorate the integrity of blood vessels and extend the hemorrhagic lesions. Neutrophils could also express inducible nitric oxide syntheses (iNOS) within 24 and 48 h, contributing to cerebrovasodilation and hemorrhage [58]. It seems that neutrophils act as an effector in the conversion between hyperperfusion and hypoperfusion during the early stage of TBI, although the underlying mechanism remains unclear.

### Neutrophils and cerebrospinal fluid (CSF)

Elevated intracranial pressure after TBI injury is mainly attributed to fluid retention in the brain and CSF cavity. The circulation and communication between cerebral capillary and CSF cavity could be significantly distorted even though the physical site of injury is remote from the ventricular system [59]. The choroid plexus epitheliums, which are also called Kolmer cells, could sense the danger/pro-inflammatory signals like interleukin-1β (IL-1β), tumor necrosis factor-α (TNF-α) released by injured brain tissue [60], and synchronize neutrophils’ trafficking as well. Neutrophils could infiltrate the choroid plexus and circulate with CSF close to the sites of injury [60, 61], which means the breakdown of blood–CSF barrier is another mechanism for neutrophils to intrude brain parenchyma. Carlos et al. reported that neutrophils were predominantly migrated from blood vessels and located at ipsilateral leptomeninges and choroid plexus within 4 h after TBI. Neutrophils were then infiltrated into the injured parenchyma and peaked within 24 to 48 h mainly through the vessels of leptomeninges and choroid plexus [62]. ICAM-1 is constitutively expressed in peripheral vessels, while platelet-endothelial adhesion molecule (PECAM-1) is exclusively expressed by choroid plexus [63], which indicates that neutrophils migrate choroid plexus endothelium with separate molecules involved. The following study also confirmed that blocking ICAM-1 or PECAM-1 could inhibit infiltration of neutrophils from peripheral vessels and choroid plexus respectively.

The components of CSF change significantly after TBI and can serve as biomarkers for the diagnosis and prognosis of TBI patients (reviewed in [64]). Several cytokines and amino acids increase dramatically in CSF, such as TNF-α, IL-6, IL-10, transforming growth factor β (TGF-β), C-X-C motif ligand chemokine 1-3 (CXCL1–3), and glutamate [25, 61, 65, 66]. Notably, these cytokines could modulate neutrophils’ function significantly. For instance, TNF-α and IL-6 promote transmigration of neutrophils; CXCL1–3 enhances chemotaxis of neutrophils; glutamate induces neutrophils’ migration by activating class I metabotropic glutamate receptors [67, 68]; TGF-β could convert neutrophils from pro-inflammatory type to anti-inflammatory type [69]. Using in vivo two-photon microscopy, Szmydynger-Chodobska et al. found that neutrophils could migrate into choroidal stroma paracellularly and reach to intercellular space between choroidal epithelial cells in rat TBI mode, jeopardizing the integrity of choroid plexus epithelium [61]. These neutrophils were accumulated at the velum interpositum within 24 h after injury and secreted vascular endothelial growth factor (VEGF) into extracellular matrix directly [70]. The secreted VEGF could circulate with CSF, exacerbating brain edema through enhancing angiogenesis and microvessel permeability. It is apparent that the CSF system could transport neutrophil-related molecules and educate neutrophils during TBI.

### Neutrophils and blood–brain barrier (BBB)

The BBB is composed of three structures: specialized endothelial cells, underlying basal membrane, and astrocytic pseudopodia. Brain endothelial cells express both endothelial and brain markers (like acetylated low density lipoprotein and gamma glutamyl transpeptidase), which make it unique compared with peripheral endothelium [71]. Of note, the expression profile of adhesion molecules in brain venules is different from peripheral tissues like post capillary venules and pulmonary capillary. For post capillary venules and pulmonary capillary, P-selectins, ICAM-1, and ICAM-2 on endothelium could be upregulated instantly at the sites of infection or injury. While in brain venules, it seems that longer time is needed for upregulation of these molecules, and this upregulation is not restricted to the damaged vessels [72]. This might determine the unique rolling, adhering and diapedesis of neutrophils in CNS during TBI compared with peripheral inflammation [71, 73]. Furthermore, the tight junction in basal membrane and pericytes around the endothelial cells also prevent specific factors and molecules from entering into parenchyma [74]. Leukocytes are usually excluded from brain by the BBB, but under pathological conditions, leukocytes can adhere to activated post-capillary venules and infiltrate the brain parenchyma across the BBB. There are two main routes for leucocytes to extricate through BBB: paracellular and transcellular. For neutrophils, transcellular pathway is the primary mechanism to migrate into brain tissue [75]. Further study indicate that neutrophils are observed only in regions exhibiting BBB damage and are initially found in injured cortex, and areas with damaged BBB within 12 h post-trauma, lining the vasculature and filling subarachnoid/subdural spaces [76]. Neutrophils then migrate from the damaged vasculature into traumatized cortical and hippocampal parenchyma by 24 h. During this process, neutrophils’ transmigration is tightly connected with activated endothelial cells, the interaction between activated neutrophils and endothelium plays an important role in secondary injury after TBI [2]. Neutrophils and endothelium could be activated by several cytokines like TNF-α, IL-1β CXCL1, CXCL2, and CXCL5 released by injured parenchyma [23, 24, 77]. Activation of endothelial cells is a decisive step in this process and can occur in vascular endothelium within minutes [78]. However, there are some differences between brain endothelium and peripheral vascular endothelium. Brain endothelium will take longer time to upregulate expression of adhesion-related molecules (ICAM-1 and VCAM-1) [72]. In addition, endothelial cell leukocytes adhesion molecule-1 (ECAM-1) is highly induced by TNF-α only in peripheral vascular endothelium but not brain endothelium [71]. The delayed activation of brain endothelium might lead to delayed neutrophils infiltration. For neutrophils migrating across the BBB from blood vessels, there are four steps involved in this process between endothelium and neutrophils: rolling, adhesion, crawling, and transmigration. In endothelium, P-selectin and E-selectin mediate the tethering and rolling of neutrophils; ICAM-1, ICAM-2, and PECAM1 participate in the adhesion and transmigration of neutrophils [62]. Inhibition of this process with ICAM1 antibody can provide benefits after injury [79, 80]. In peripheral tissues, expression of P-selectin, glycoprotein ligand 1 and CD11a/CD18 on neutrophils are vital for rolling [81] while CXCR1 and CXCR2 are indispensable for the binding to the endothelium [30]. Expression of CXCR2 ligands, such as CXCL1, CXCL2, and CXCL5, can force invasion of neutrophils into the CNS parenchyma. There are some benefits for TBI with P-selectin blocking [82–84] as well as CXCR2 and CXCR4 inhibitors [85, 86]. Other than transmigration, neutrophils also damage the tight junction and permeability of BBB simultaneously. For example, leukocytes recruitment leads to degradation and redistribution of occludin, zonula occludens-1, β-catenin, and vinculin, which are vital for the integrity of BBB. Neutrophil-released NE significantly disrupts the cadherin–cadherin binding, causing hyperpermeability of BBB [87, 88]. Neutrophil-derived MMPs also play vital roles in dysregulation of blood–brain barrier after TBI, including MMP2, MMP3, and MMP9 [89, 90]. These MMPs can be significantly induced by IL-1β within 24 h and degrade structures of the neurovascular unit after injury. Blocking the activity of MMP9 by related drugs [91–94], or knocking down expression of the MMP9, shows some protections for BBB in acute phase [95]. Activated neutrophils are also the pivotal source of free radicals like reactive oxygen species (ROS), nitrous oxide (NOS), and NADPH oxidase. These free radicals induce direct oxidative damage, rearrangement of claudin-5 and occludin in endothelium via PI3K-AKT pathways, contributing to the break-up of the integrity of BBB [96, 97]. ROS or NO inhibitor could improve the integrity of BBB after injury [98, 99]. In addition to the effect from the above neutrophil-derived cytokines, the formation of NETs could directly lead to epithelial and endothelial cell death [100]. Obviously, breakdown of BBB by the neutrophils shall help to recruit more immune cells to fight against pathogens; this also leads to indiscriminate tissue damage at the same time. Strategies to inhibit activated neutrophils or improve the stability of BBB shall prove to be beneficial for the inflammatory resolution and the following recovery.

### Neutrophils and edema

Cerebral edema is the result of excess accumulated fluid in the intracellular or extracellular spaces of the brain, leading to expansion of brain tissue in limited skull cavity. It is a vital pathogenic impact since it increases intracranial pressure, impairs cerebral perfusion and oxygenation, and contributes to additional ischemic injuries after TBI [101]. There are three major types of edema after TBI [102]: vasogenic edema, cytotoxic edema, and osmotic edema. Vasogenic edema is due to a breakdown of BBB, which allows intravascular proteins and fluid to penetrate into the parenchyma extracellular space. Cytotoxic edema is the result of abnormal water collection in the cells. Osmotic edema is caused by osmotic imbalances between blood and tissue. Neutrophils and edema are tightly connected after trauma and several studies show that depletion of neutrophils significantly decreases tissue edema [43, 82, 92, 103, 104]. Since the vasogenic edema is tightly connected with vascular integrity of BBB, the interaction between neutrophils and BBB could influence vasogenic edema as well. As discussed above (see the “Neutrophils and blood–brain barrier (BBB)” section), neutrophils are involved in the breakdown of BBB through the abnormal interactions between endothelium and neutrophils. Despite the chemokines, proteases, and free radicals released by injured tissue or neutrophils, various mediators could enhance vasogenic and cytotoxic edema, such as glutamate, lactate, H^+^, K^+^, Ca^2+^, histamine, and kinins, and could also promote extensive neutrophils infiltration and neuronal-astroglial loss as well [105].

Cytotoxic edema is usually accompanied by the accumulation of Na^+^ and other cations intracellularly. Extracellular Na^+^, Cl^−^ and water enter into cells and create a new gradient for these molecules to move across the capillary of BBB [106]. Na^+^/H^+^ exchanger is an important channel that modulates this process [107]. Over engulfment of Na^+^ by the cells leads to the H^+^ accumulation extracellularly, facilitating the activation of neutrophils in a leukotriene B_4_ dependent manner [108]. Suzuki et al. showed that inhibition of Na^+^/H^+^ exchanger with SM-2022 could significantly attenuate cerebral infarct volume, water content, and the neutrophils accumulation in ischemia model [107]. Thus, apart from traditional cytokines, both the acidity and cations in the injured brain tissue could modulate activation of neutrophils and cytotoxic edema. It is noteworthy that BBB breakdown and cell swelling are not the only reason for brain edema after injury. The osmotic imbalance between blood and tissue is another mechanism for brain edema. For instance, hyponatremia, which is a common feature of clinical TBI, could cause osmotic edema. Hyperperfusion or hypoperfusion is also crucial for the osmotic edema as well, whose relationships with neutrophils have been discussed in the “Neutrophils and blood flow” section. The permeability of blood vessel could also modulate osmotic edema. The neutrophil-released granules or particle-like elastase, azurocidin, and lipoxin are all tightly connected with the vascular permeability in the peripheral tissues [109–111]. The connection between neutrophil infiltration and brain edema seems to be a time-dependent issue. Depletion of neutrophils decreases the tissue edema from injury after 24–48 h or longer time [47, 112, 113]. On the other hand, depletion of neutrophils within 4 h after TBI showed no impact on edema [104]. Some people also claimed that there is no direct relationship between brain edema and leukocytes accumulation [82]. More work needs to be done to better understand when and how neutrophils influence edema after TBI.

### Neutrophils and hypoxia

The imbalance between cerebral oxygen delivery and consumption is another characteristic of TBI [2, 32]. The changes in the volume and flow rate of blood and arterial oxygen content after injury could instantly compromise the oxygen delivery, leaving the injured area under a hypoxic condition for both brain resident cells as well as the infiltrated neutrophils. The duration and extent of hypoxia are correlated with the outcomes of TBI patients [114]. The deprivation of oxygen after TBI may also occur even though the patients have the normal cerebral perfusion pressure or blood oxygen saturation [115]. A previous study showed that low levels of oxygen and glucose induce the expression of hypoxia-inducible factor -1α and NFκB, leading to prolongation of neutrophils’ survival time and their activation simultaneously [116]. Activated neutrophils could consume more oxygen to generate and release superoxide hydrogen peroxide to maintain its phagocytic functions [117, 118], accompanied with overt release of NADPH oxygenase-related molecules (ROS) [97, 119] and antibacterial proteins (cathelicidin and defensins) [120, 121]. Meanwhile, neutrophils could induce over-production of ROS themselves in an IL-17 autocrine manner, aggravating ROS related injury [122]. It is possible that neutrophils compete with brain cells to consume more oxygen to maintain their activated status and lead to uncontrolled damage. Several experiments reported that hypoxia could induce microglia to release nitric oxide (NO) and a series of cytokines to maintain the activation of neutrophils, leading to persistent hypoxia and neuronal death [123, 124]. In the clinic, patients with head trauma are frequently associated with respiratory problems, like lung injury, pneumonia, or respiratory distress syndrome, resulting in secondary hypoxic insult as well as overwhelming activation of neutrophils [125]. Hypoxia is an important factor that promotes and maintains the over production of free radicals from neutrophils after TBI, blockade of degranulation, and oxidative burst of neutrophils has shown benefits for brain injury [98].

### Neutrophils and neuroinflammation

Neuroinflammation is the inflammatory responses in CNS, where brain resident cells and peripheral immune cells participate together. Although the response is initiated to protect the CNS from infection and damage, it is also an important mechanism to induce secondary injury after TBI. TBI-related neuroinflammation is characterized by activated glia, recruited leucocytes, and upregulated inflammatory cytokines in the brain [126].

Glia in CNS consists of microglia, oligodendrocyte, and astrocytes, all of which are indispensable to maintain homeostasis in the brain and support normal neuronal functions [127]. Microglia is innate immune cell of the brain, which is analogous to macrophage in peripheral tissue. It functions in scavenging debris, surveying circumambient microenvironment and transferring inflammatory signals. There are two states of microglia: quiescent or priming. The quiescent microglia can be induced to priming microglia by stroke, aging, brain injury, and neurodegenerative disease. In addition to the two states, there are two subpopulations in microglia based upon their functions. The subtype associated with phagocytosis and capacity to kill pathogens is called M1; the one that is involved in tissue repair and growth stimulation is called M2. So far, there is no direct evidence to indicate that neutrophils influence the subpopulation (M1 and M2) of microglia in TBI model. However Moxon-Emre and Schlichter showed that depletion of neutrophils could reduce microglia/macrophage populations after intracerebral hemorrhage, as well as decrease the activation marker CD68 on microglia/macrophage, indicating that neutrophils participate in modulating microglia state [128]. The resident microglia senses the change of damage-associated molecular patterns as well as pathogen-associated molecular patterns. The primed microglia could initiate the activation of endothelial cells as well as the recruitment of peripheral leukocytes into the CNS [129]. The activated microglia rapidly produces large amounts of inflammatory cytokines and chemokines (IL-1β, TNF-α, IL-6, CXCL1–5, CXCL8–10), as well as rearranges patterns of receptor expressions (major histocompatibility complex II and complement receptor 3) [130]. These inflammatory mediators are strong chemokines to recruit and activate neutrophils. In addition to the above microglia-derived cytokines, neutrophils release some other molecules to activate microglia reciprocally, such as ROS [131], lipocalin 2 [132], and MMP9 [133]. The activation of microglia and neutrophils is a cascade amplification. Interestingly, the activated microglia behaves like a double-edged sword: on the one hand, it promotes neutrophils to secrete more pro-inflammatory cytokines to form the cytokine storm; on the other hand, the activated microglia is also correlated with the reduction of neuronal damage, releasing microglia neuroprotective factors [134]. Some of microglia secreted cytokines are also beneficial for nerve recovery (listed in the Table 1). Total ablation of microglial cells results in a significant increase in the infracted size and the number of apoptotic neurons [135]. The interactions between quiescent microglia and neutrophils are quite different. The inactivated microglia generally engulf and scavenge the motile and robust neutrophils in an α_v_β_3_-integrin and lectin-like receptor-dependent manner, protecting the neurons from damage [136]. It is unclear if neutrophils could influence microglia M1/M2 polarization in the settings of severity, location, and period of injury.

Mature oligodendrocytes wrap around axons or blood vessels, forming the myelin sheath of nerve insulation structure and maintaining the normal functions of neurons. They are classified into three sorts based upon the distribution: interfascicular oligodendrocytes, perineuronal oligodendrocytes, and perivascular oligodendrocytes. The interfascicular oligodendrocytes are distributed along the white matters and nerve fibers, which is rapidly reduced within the phase of myelin formation. The perineuronal oligodendrocytes, also called perineuronal satellite cells, can be proliferated from oligodendrocyte precursor cells (OPCs) as a response to injury or disease. They are thought to maintain homeostasis and participate in post-injury repair. OPCs are known to constitute the majority of MMP9-expressing cells in the acute phase of brain injury, and standard MMP inhibitor GM6001 reduced the early BBB leakage and neutrophils infiltration [137]. Oligodendrocytes also secrete significant amounts of CXC chemokines, such as CXCL1–5 and CCL21 to induce neutrophils invasion and astrogliosis [24, 138, 139], which indicates that oligodendrocytes might collaborate with neutrophils to aggravate inflammation. Interestingly, neutrophils could damage oligodendrocytes as well. Mice that are deficient in CXCR2, the predominant receptor for recruiting neutrophils, are more resistant to demyelination after injury. Specifically, CXCR2^+^ neutrophils are the main driving force to cause oligodendrocytes loss and demyelination, influencing the myelination of damage axon and long-term recovery after TBI [140]. There are limited studies about the relationship between perivascular oligodendrocytes and neutrophils. Whether perivascular oligodendrocytes interact with endothelium to facilitate neutrophil’s transmigration is an interesting research direction.

Astrocytes are the amplest glia cell in CNS, which indispensably maintain the CNS homeostasis and contribute to the integrity of BBB with perivascular cells. Astrocytes usually exhibit a pathological process termed as “reactive astrogliosis” in response to CNS injury, contributing to CNS neuropathology [141]. However, the role of astrogliosis in injury recovery remains equivocal. Under injury conditions, neutrophils and astrocytes are tightly connected each other and respond to shared cytokines. Astrocytes are vital cytokine source that provides several cytokines and proteinases, like IL-6, CCL2, CXCL1, CXCL2, GM-CSF, glutamate-leucine-arginine motif containing chemokines, MMP2 and MMP9 [142–144]. Some cytokines promote BBB disruption, leukocytes recruitment, and inflammation initiation such as CXCL1, CXCL2, and GM-CSF [141, 145–148], while others are essential for the astrocytes and neurons’ protection like IL-6 and CCL2 [66, 149]. These cytokines are also important for modulating glutamate uptake by astrocytes, which is considered as a protective mechanism during neuroinflammation. IL-1β and TNF-α inhibit astrocytes glutamate uptake in a dose-dependent manner, whereas interferon-γ alone stimulates this activity [150]. Meanwhile, the reactive astrocytes could form perivascular scars as well that restricts the spread of neutrophils from the damaged tissue into healthy parenchyma during the acute inflammatory responses [151]. In an ex vivo experiment, Xie et al. showed dependent on the way (direcet vs indirect) neutrophils interact with astrocytes, the outcome can be different [152]. Under direct cell-cell contact, astrocytes could decrease the apoptosis, respiratory burst, and degranulation of neutrophils, enhancing neutrophils phagocytic capability and pro-inflammatory cytokine expression. Under indirect interaction, astrocytes attenuate apoptosis and enhance necrosis in neutrophils, augment neutrophils phagocytosis and respiratory burst, and inhibit neutrophils degranulation [152]. Reciprocally neutrophils can influence the evolution of astrocytes reactivity as well. Treatment of mice with anti-Ly6G antibody dampened the astrogliosis and worsened the behavioral outcomes in spinal cord injury [113]. In another in vitro study, Hooshmand and Nguyen showed that neutrophils could induce astrogliogenesis via generating C1q and C3a [153]. All of these data suggest that neutrophils and astrocytes work as the main source of cytokines during neuroinflammation, boosting the inflammation cascade with mutual stimulation. Neutrophils are definitely an important driver to promote reactive astrogliosis during injury. Whether astrogliosis is good or bad in brain injury remains an open question.

### Neutrophils and neurodegeneration

A retrospective study showed that the neuroinflammation and white matter degeneration would persist for several years after TBI [154]. The TBI patients are more vulnerable to develop dementia, Alzheimer’s disease (AD), and Parkinson’s disease (PD) [154, 155]. In the animal model, neutrophils seem to be involved in white matter inflammation and degeneration [5, 156]. Neutrophils could also drive initiation of the AD-like symptoms [157] and participate in the formation of Aβ plaques [158]. These results highlight the fact that neutrophils function beyond the acute phase and work with other innate immune cells to contribute to the pathogenesis of degenerative diseases after TBI. Several acute inflammatory cytokines, like IL-1β, ROS, and complement 5a (C5a), are all shown to be tightly related to neurodegeneration [159–161]. There is an elevated level of oxidative stress in neutrophils isolated from AD and PD patients [160]. The author speculated that the overwhelmed reactive species might influence the mitochondrial electron membrane of CNS cells. In return, the degenerative substances like beta-amyloid could also stimulate neutrophils to produce more oxidative substances. However, some research suggest that there is no direct relationship between neutrophils and degeneration [162]. In that study, the authors found that neutrophils were observed in the parenchyma 1 year after the induction, indicating that neutrophils might survive for a long-term period in CNS environment [162]. But there is no evidence of neuronal degeneration in the tissue. Neutrophils might modulate the degeneration indirectly with other immune cells like Treg cells, Th17 cells, and ϒδ T cells [6]. Intriguingly, a recent investigation indicated that the accumulation of amyloid-beta peptide might be an antimicrobial peptide to fight against inherent brain infection [163], which could be interpreted that AD might be an infectious disease. It is well-known that brain trauma usually causes open injury in the tissue, leaving the possibility of concomitant infection for a lifelong time. Whether neutrophils would collaborate with the amyloid-beta peptide to fight against infection is another interesting and promising direction for the future.

### Neutrophils and nerve recovery

It is clear that infiltrated neutrophils cause brain tissue damage, especially to neurons. Neutrophils were shown to affect the survival of neurons directly in a neutrophils-neurons coculture study: the neutrophils caused the neurons’ death through direct contact and released proteases, like MMP9 and NE [57, 164–166]. However, it is unclear whether the infiltrated neutrophils have preference for neurons. In other words, is it possible that damaged neurons positively attract neutrophils? Chung-ha et al. and Hayakawa et al. confirmed that neurons could exchange signalings with astrocytes by releasing mitochondria [167, 168]. Leow-Dyke et al. showed that neurons could release a cluster of cytokines (CXCL1, TNF-α, and IL-6) to promote neutrophils’ infiltration across the endothelial monolayer [169]. These neuron-released substances might also be potential “helping signal” in the CNS. Neutrophils are actively recruited to the injured brain tissue, where they clean up debris, protect against potential infection of the exposed parenchyma, and promote tissue regeneration [170]. With visualization by two-photon microscopy in a TBI model, Roth et al. revealed that infiltrated neutrophils were primarily swarmed in the damaged meningeal area and interacted with dead cells and debris. Blocking this process led to increased amounts of cell death in the meninges, suggesting that at least in meningeal space, neutrophils play a protective role [171]. Kurimoto et al. confirmed that neutrophils promoted retinal ganglion cells to regenerate lengthy axons in injured optic nerve. The influx of neutrophils started to appear in the mouse eyes within hours after injury as well as the elevated expression levels of atypical growth factor oncomodulin (Ocm). Depletion of neutrophils diminished the Ocm and abolished the pro-regenerative effects of inflammation at the same time [172]. TGF-β is another factor that neutrophils could secret after stimulation [173]. It could promote nerve regeneration by stimulating nerve growth factor production as well as controlling the astrocytosis and scar formation after injury [174]. Neutrophils could also secrete several other molecules that are beneficial for promoting neuron cell survival, like G-CSF, hepatocyte growth factor (HGF), nerve growth factor (NGF), and neurotrophin-4 (NT4) [28, 175–178]. Several cytokines that modulate neutrophils’ function also show the effects of augmenting neurite growth as well as preventing neuronal cell death, such as arginase and midkine [26, 179]. Angiogenesis is an essential process for damaged brain tissue to restore perfusion after TBI. Of note, neutrophils themselves are a source of VEGF to modulate their migration and contribute to angiogenesis in an autocrine manner [180]. The newborn blood vessels could provide mediators that are critical for neurogenesis at injury sides such as brain-derived neurotrophic factor (BDNF) and VEGF [27]. In general, chemokines that promote neutrophil infiltration (some of the chemokines are also produced by themselves) also promote angiogenesis, such as CXCL1, CXCL8, and granulocyte chemotactic protein-2 [181, 182], MMP8 and MMP9 [183], all of which are tightly connected with vessel growth and remodeling [184, 185]. Finally, increasing evidences suggest that neutrophils could also disseminate anti-inflammatory microparticles and participate in inflammation resolution [186]. Several cytokines produced by neutrophils can also promote resolution of inflammation, including IL-4, IL-10, and TGF-β [187, 188]. It is still unknown whether neutrophils’ action contributes to nerve repair or exacerbate inflammation.

## Discussions

Neutrophils have long been believed to be short-lived immune cells and contribute to brain damage during the acute phase of brain injury. Elimination of neutrophils or inhibition of neutrophils recruitment show some protective effects in brain injury [83, 84, 104, 189]. However, inhibition of neutrophils in the clinic might carry a significant risk of severe hospitalized infections and immunologic dissonance [190–192]. Furthermore, existing evidence does not fully support the notion that more neutrophils’ recruitment leads to the more severe brain injury [46, 162, 165, 171, 172, 193]. Recently, the development and application of advanced neurotechnologies have helped researchers to gain deep insight into the roles of neutrophils in CNS injury and other diseases, which challenges our conventional views on neutrophils and call for more studies on many new questions in the future. Listed below are four important questions that deserve attention in the near future:

1. *Can neutrophils be divided into two subpopulations?* Several studies suggest the presence of two subpopulations of neutrophils with opposite roles, similar to microglia and macrophage (M1 and M2). In tumor models, neutrophils can be converted from anti-tumor phenotype (N1: release ROS and TNF-α) to pro-tumor phenotype (N2: increased Arginase, CCL2, and CCL5) by the TGF-β stimulation [194]. In infection models, there are also two distinct subpopulations of neutrophils: pro-inflammatory neutrophils (N1: IL-12 and macrophage inflammatory proteins producing) and anti-inflammatory neutrophils (N2: IL-10 and CCL2 producing) [195]. In a stroke model, it was shown that rosiglitazone, one of the agonists for peroxisome proliferator-activated receptor-γ, could promote the infiltration of N2-like neutrophils into the ischemic core and protect neuron damage concomitantly through facilitating the dissolution of inflammatory responses [196]. This study proves that N2 phenotype of neutrophils is beneficial for the brain injury. Despite the compelling evidence for the separate subsets of neutrophils, there are no specific markers to identify and distinguish them, which warrants more investigation in the future. Their roles in the CNS diseases may depend on pro/anti-inflammatory (N1, N2) phenotypes that are regulated by specific environmental cues in the brain after injury. Further understanding of these cues and the outcomes associated with particular phenotypes may allow neutrophils to serve as disease-modifying factors in the CNS.

2. *How long will neutrophils survive?* Latest studies raise another important question about the average lifespan of neutrophils under normal and disease conditions. It has long been regarded that the average half lifespan of neutrophils in circulation is around 1.5 and 8 h in mice and human, respectively. The exact half-life of human neutrophils varies from 7 to 22 h in different disease conditions and labeling methods (reviewed in [197]). Recent studies indicated that the average lifespan of human neutrophils in circulation could be as long as 5.4 days [198]. The time window that neutrophils might play in TBI may be significantly longer than previously thought since specific conditions in the brain could prolong the survival time of neutrophils like hypoxia [116], glutamate [199], ATP, and adenosine [200, 201]. In addition, due to the damage of BBB after TBI, circulating neutrophils still can continuously transmigrate into brain tissue even though the damaged tissue start to gradually recover from TBI. Data from previous studies of other groups and our recent work (unpublished) show that neutrophils can be detected in damaged brain tissue after 14 days or even 1 year later [162]. Thus the real function of neutrophils in TBI is likely to go beyond fighting against pathogens in the acute phase. A role of neutrophils in modulating the activation of T cells and B cells in chronic phase should also be taken into consideration [202, 203].

3. *Is the recruitment cascade of neutrophils in the brain similar to that in peripheral tissues?* The recruitment of neutrophils involves several steps including tethering, rolling, adhesion, crawling, and transmigration [30]. Previous studies show that P-selectin and E-selectin mediate the tethering and rolling of neutrophils; ICAM-1, ICAM-2, and PECAM1 participate in the adhesion and transmigration of neutrophils [62]. However, it appears that rolling and migration of neutrophils in the brain venule are governed by a different mechanism, as brain endothelium (BE) differs from peripheral endothelium (PE) at least three aspects: (1) adhesion molecules are different (BE:ICAM-1^high^, VCAM-1^high^, ECAM-1^low^; PE:ICAM-1^high^, VCAM-1^high^, ECAM-1^high^) [71]; (2) activation times are different (BE: 2–48 h; PE: within minutes) [72]; and (3) existence of pericytes and astrocytes around BE [204, 205]. The specific molecules involved in adhesion and transmigration of neutrophils in brain venules are currently unknown and require more studies in the future [30].

4. *Is it possible that the infiltrated neutrophils could be reprogammed and return to peripheral in TBI?* The infiltrated neutrophils could be reprogrammed by the brain resident cells or molecules. Glutamate, as the essential neurotransmitter in the brain, could enhance migration, promote immune responses, and prolong survival time of neutrophils [67, 199, 206]. Astrocytes, as the amplest glia in the brain, could also modulate neutrophils’ functions directly and indirectly [152]. Previous studies have also shown that neutrophils could be reprogrammed into specific phenotype with augmented phagocytic index [196, 207]. Since the reprogrammed neutrophils have enhanced capacity of cell killing and degranulation, they might induce more severe tissue damage if they move back to peripheral circulation. Recently, some studies showed that neutrophils might migrate back to vessel from infiltrated tissue, which is also called reverse migration [208, 209]. This phenomenon not only provides a novel mechanism of inflammation resolution but also provides a possibility of inducing secondary damage in peripheral organs when they migrate back to circulation. In severe TBI patients, the acute respiratory injury and multiple organ failures are quite common, which is tightly connected with neutrophils’ function [210, 211]. Thus, we speculate that injured brain tissue might re-educate neutrophils to become a pro-inflammatory and long-survival subtype. Upon return back to circulation, these re-educated neutrophils can cause damages to the distant peripheral organs/tissues.

## Conclusions

Neutrophils are a crucial component of the innate immune system, whose inappropriate or excessive activation could lead to tissue damage. TBI-induced changes in immune system play a decisive role in its development and prognosis. The phenotype, function, and survival time of neutrophils are tightly connected with every pathological process of TBI, including changes in blood flow and CSF component, breakdown of BBB, alterations in brain metabolism, neuroinflammation, neurodegeneration, and nerve recovery. The plasticity of neutrophils and crosstalk with other cells also complicate their functions in TBI. Thus, it is hasty to conclude that neutrophils function in a well-defined and limited manner since the roles of neutrophils of being protective or harmful depend on the phase and type of insult, and the type of cells neutrophils are interacting with. Literature indicates that the different microenvironments contribute to the different features of neutrophils in the brain compared with those in peripheral tissues under inflammatory conditions. However, the molecular mechanisms of how the brain environment regulates neutrophils’ functions and how the neutrophils’ functions affect brain injury and repair have not been well elucidated. More studies are needed to better define the complex roles of neutrophils at different stages of TBI as well as the underlying mechanisms. It is possible that a new and improved therapeutic strategy will be developed in the future that will selectively eliminate the harmful effect of neutrophils while keeping their beneficial effect in TBI.

## Abbreviations

AD: Alzheimer’s disease
BBB: Blood-brain barrier
BDNF: Brain-derived neurotrophic factor
BE: Brain endothelium
CBF: Cerebral blood flow
CCL: C-C motif ligand chemokine
CNS: Central nervous system
CSF: Cerebrospinal fluid
CXCL: C-X-C motif ligand chemokine
CXCR: C-X-C chemokine receptor
ECAM: Endothelial cell adhesion molecule
G-CSF: Granulocyte-colony stimulating factor
GM-CSF: Granulocyte-macrophage-colony stimulating factor
HGF: Hepatocyte growth factor
ICAM: Intercellular adhesion molecule
IL: Interleukin
iNOS: Inducible nitric oxide syntheses
MMPs: Matrix metalloproteinases
NE: Neutrophils elastase
NETs: Neutrophils extracellular traps
NFκB: Nuclear factor kappa-light-chain-enhancer of activated B cells
NGF: Nerve growth factor
NOS: Nitrous oxide
NT4: Neurotrophin-4
Ocm: Oncomodulin
OPCs: Oligodendrocyte precursor cells
PD: Parkinson’s disease
PE: Peripheral endothelium
PECAM: Platelet-endothelial adhesion molecules
PMNs: Polymorph nuclear cells
ROS: Reactive oxygen species
TBI: Traumatic brain injury
TGF: Transforming growth factor
TNF: Tumor necrosis factor
VCAM: Vascular cell adhesion molecule
VEGF: Vascular endothelial growth factor

## References

[1] Hazeldine J, Lord JM, Belli A (2015). Traumatic brain injury and peripheral immune suppression: primer and prospectus. Front Neurol.

[2] Werner C, Engelhard K (2007). Pathophysiology of traumatic brain injury. Br J Anaesth.

[3] Kim E, Lauterbach EC, Reeve A, Arciniegas DB, Coburn KL, Mendez MF, Rummans TA, Coffey EC (2007). Neuropsychiatric complications of traumatic brain injury: a critical review of the literature (a report by the ANPA Committee on Research). J Neuropsychiatr Clin Neurosci.

[4] Hazeldine J, Hampson P, Lord JM (2014). The impact of trauma on neutrophil function. Injury.

[5] Donnelly DJ, Popovich PG (2008). Inflammation and its role in neuroprotection, axonal regeneration and functional recovery after spinal cord injury. Exp Neurol.

[6] Mantovani A, Cassatella MA, Costantini C, Jaillon S (2011). Neutrophils in the activation and regulation of innate and adaptive immunity. Nat Rev Immunol.

[7] Morganti-Kossmann MC, Rancan M, Stahel PF, Kossmann T (2002). Inflammatory response in acute traumatic brain injury: a double-edged sword. Curr Opin Crit Care.

[8] Ermert D, Niemiec MJ, Rohm M, Glenthoj A, Borregaard N, Urban CF (2013). Candida albicans escapes from mouse neutrophils. J Leukoc Biol.

[9] Akashi K, Traver D, Miyamoto T, Weissman IL (2000). A clonogenic common myeloid progenitor that gives rise to all myeloid lineages. Nature.

[10] Wilson EH, Weninger W, Hunter CA (2010). Trafficking of immune cells in the central nervous system. J Clin Invest.

[11] Khajah M, Millen B, Cara DC, Waterhouse C, McCafferty DM (2011). Granulocyte-macrophage colony-stimulating factor (GM-CSF): a chemoattractive agent for murine leukocytes in vivo. J Leukoc Biol.

[12] Beyrau M, Bodkin JV, Nourshargh S (2012). Neutrophil heterogeneity in health and disease: a revitalized avenue in inflammation and immunity. Open Biol.

[13] da Silva FM, Massart-Leën A, Burvenich C (1994). Development and maturation of neutrophils. Vet Q.

[14] Cowland JB, Borregaard N (1999). The individual regulation of granule protein mRNA levels during neutrophil maturation explains the heterogeneity of neutrophil granules. J Leukoc Biol.

[15] Martin C, Burdon PC, Bridger G, Gutierrez-Ramos JC, Williams TJ, Rankin SM (2003). Chemokines acting via CXCR2 and CXCR4 control the release of neutrophils from the bone marrow and their return following senescence. Immunity.

[16] Kjeldsen L, Cowland JB, Johnsen AH, Borregaard N (1996). SGP28, a novel matrix glycoprotein in specific granules of human neutrophils with similarity to a human testis-specific gene product and a rodent sperm-coating glycoprotein. FEBS Lett.

[17] Min H, Hong J, Cho IH, Jang YH, Lee H, Kim D, Yu SW, Lee S, Lee SJ (2015). TLR2-induced astrocyte MMP9 activation compromises the blood brain barrier and exacerbates intracerebral hemorrhage in animal models. Mol Brain.

[18] Fortin CF, Ear T, McDonald PP (2009). Autocrine role of endogenous interleukin-18 on inflammatory cytokine generation by human neutrophils. FASEB J.

[19] Surette ME, Krump E, Picard S, Borgeat P (1999). Activation of leukotriene synthesis in human neutrophils by exogenous arachidonic acid: inhibition by adenosine A2a receptor agonists and crucial role of autocrine activation by leukotriene B4. Mol Pharmacol.

[20] Au B, Williams TJ, Collins PD (1994). Zymosan-induced IL-8 release from human neutrophils involves activation via the CD11b/CD18 receptor and endogenous platelet-activating factor as an autocrine modulator. J Immunol.

[21] Keeling K, Hicks R, Mahesh J, Billings B, Kotwal G (2000). Local neutrophil influx following lateral fluid-percussion brain injury in rats is associated with accumulation of complement activation fragments of the third component (C3) of the complement system. J Neuroimmunol.

[22] Barkalow F, Goodman M, Gerritsen M, Mayadas T (1996). Brain endothelium lack one of two pathways of P-selectin-mediated neutrophil adhesion. Blood.

[23] Aloisi F, Care A, Borsellino G, Gallo P, Rosa S, Bassani A, Cabibbo A, Testa U, Levi G, Peschle C (1992). Production of hemolymphopoietic cytokines (IL-6, IL-8, colony-stimulating factors) by normal human astrocytes in response to IL-1 beta and tumor necrosis factor-alpha. J Immunol.

[24] Johnson EA, Dao TL, Guignet MA, Geddes CE, Koemeter-Cox AI, Kan RK (2011). Increased expression of the chemokines CXCL1 and MIP-1alpha by resident brain cells precedes neutrophil infiltration in the brain following prolonged soman-induced status epilepticus in rats. J Neuroinflammation.

[25] Csuka E, Morganti-Kossmann MC, Lenzlinger PM, Joller H, Trentz O, Kossmann T (1999). IL-10 levels in cerebrospinal fluid and serum of patients with severe traumatic brain injury: relationship to IL-6, TNF-alpha, TGF-beta1 and blood-brain barrier function. J Neuroimmunol.

[26] Fenn AM, Hall JCE, Gensel JC, Popovich PG, Godbout JP (2014). IL-4 signaling drives a unique arginase(+)/IL-1β(+) microglia phenotype and recruits macrophages to the inflammatory CNS: consequences of age-related deficits in IL-4Rα after traumatic spinal cord injury. J Neurosci.

[27] Chen J, Zhang C, Jiang H, Li Y, Zhang L, Robin A, Katakowski M, Lu M, Chopp M (2005). Atorvastatin induction of VEGF and BDNF promotes brain plasticity after stroke in mice. J Cereb Blood Flow Metab.

[28] Royo N, Conte V, Saatman K, Shimizu S, Belfield C, Soltesz K, Davis J, Fujimoto S, McIntosh T (2006). Hippocampal vulnerability following traumatic brain injury: a potential role for neurotrophin-4/5 in pyramidal cell neuroprotection. Eur J Neurosci.

[29] Bennett G, Al-Rashed S, Hoult J, Brain S (1998). Nerve growth factor induced hyperalgesia in the rat hind paw is dependent on circulating neutrophils. Pain.

[30] Kolaczkowska E, Kubes P (2013). Neutrophil recruitment and function in health and inflammation. Nat Rev Immunol.

[31] Williams OH, Tallantyre EC, Robertson NP (2015). Traumatic brain injury: pathophysiology, clinical outcome and treatment. J Neurol.

[32] Werner JK, Stevens RD (2015). Traumatic brain injury: recent advances in plasticity and regeneration. Curr Opin Neurol.

[33] Louveau A, Smirnov I, Keyes TJ, Eccles JD, Rouhani SJ, Peske JD, Derecki NC, Castle D, Mandell JW, Lee KS (2015). Structural and functional features of central nervous system lymphatic vessels. Nature.

[34] Johnson VE, Stewart W, Smith DH (2013). Axonal pathology in traumatic brain injury. Exp Neurol.

[35] Coles JP, Fryer TD, Smielewski P, Rice K, Clark JC, Pickard JD, Menon DK (2004). Defining ischemic burden after traumatic brain injury using 15O PET imaging of cerebral physiology. J Cereb Blood Flow Metab.

[36] Inoue Y, Shiozaki T, Tasaki O, Hayakata T, Ikegawa H, Yoshiya K, Fujinaka T, Tanaka H, Shimazu T, Sugimoto H (2005). Changes in cerebral blood flow from the acute to the chronic phase of severe head injury. J Neurotrauma.

[37] Stein DM, Hu PF, Brenner M, Sheth KN, Liu KH, Xiong W, Aarabi B, Scalea TM (2011). Brief episodes of intracranial hypertension and cerebral hypoperfusion are associated with poor functional outcome after severe traumatic brain injury. J Trauma.

[38] Kelly DF, Martin NA, Kordestani R, Counelis G, Hovda DA, Bergsneider M, McBride DQ, Shalmon E, Herman D, Becker DP (1997). Cerebral blood flow as a predictor of outcome following traumatic brain injury. J Neurosurg.

[39] Worthen GS, Schwab B, Elson EL, Downey GP (1989). Mechanics of stimulated neutrophils: cell stiffening induces retention in capillaries. Science.

[40] Yang L, Froio RM, Sciuto TE, Dvorak AM, Alon R, Luscinskas FW (2005). ICAM-1 regulates neutrophil adhesion and transcellular migration of TNF-alpha-activated vascular endothelium under flow. Blood.

[41] Walcheck B, Moore KL, McEver RP, Kishimoto TK (1996). Neutrophil-neutrophil interactions under hydrodynamic shear stress involve L-selectin and PSGL-1. A mechanism that amplifies initial leukocyte accumulation of P-selectin in vitro. J Clin Invest.

[42] Roca-Cusachs P, Almendros I, Sunyer R, Gavara N, Farre R, Navajas D (2006). Rheology of passive and adhesion-activated neutrophils probed by atomic force microscopy. Biophys J.

[43] Uhl MW, Biagas KV, Grundl PD, Barmada MA, Schiding JK, Nemoto EM, Kochanek PM (1994). Effects of neutropenia on edema, histology, and cerebral blood flow after traumatic brain injury in rats. J Neurotrauma.

[44] Zhang X, Cheng R, Rowe D, Sethu P, Daugherty A, Yu G, Shin HY (2014). Shear-sensitive regulation of neutrophil flow behavior and its potential impact on microvascular blood flow dysregulation in hypercholesterolemia. Arterioscler Thromb Vasc Biol.

[45] Cohen MJ, Brohi K, Ganter MT, Manley GT, Mackersie RC, Pittet JF (2007). Early coagulopathy after traumatic brain injury: the role of hypoperfusion and the protein C pathway. J Trauma.

[46] Emerich DF, Dean RL, Bartus RT (2002). The role of leukocytes following cerebral ischemia: pathogenic variable or bystander reaction to emerging infarct?. Exp Neurol.

[47] Palmer C, Roberts RL, Young PI (2004). Timing of neutrophil depletion influences long-term neuroprotection in neonatal rat hypoxic-ischemic brain injury. Pediatr Res.

[48] Kelly DF, Kordestani RK, Martin NA, Nguyen T, Hovda DA, Bergsneider M, McArthur DL, Becker DP (1996). Hyperemia following traumatic brain injury: relationship to intracranial hypertension and outcome. J Neurosurg.

[49] Adelson PD, Clyde B, Kochanek PM, Wisniewski SR, Marion DW, Yonas H (1997). Cerebrovascular response in infants and young children following severe traumatic brain injury: a preliminary report. Pediatr Neurosurg.

[50] Bouma GJ, Muizelaar JP, Bandoh K, Marmarou A (1992). Blood pressure and intracranial pressure-volume dynamics in severe head injury: relationship with cerebral blood flow. J Neurosurg.

[51] Issekutz AC (1981). Vascular responses during acute neutrophilic inflammation. Their relationship to in vivo neutrophil emigration. Lab Investig.

[52] Thompson HJ, Tkacs NC, Saatman KE, Raghupathi R, McIntosh TK (2003). Hyperthermia following traumatic brain injury: a critical evaluation. Neurobiol Dis.

[53] Farooq MU, Goshgarian C, Min J, Gorelick PB (2016). Pathophysiology and management of reperfusion injury and hyperperfusion syndrome after carotid endarterectomy and carotid artery stenting. Exp Transl Stroke Med.

[54] Kurland D, Hong C, Aarabi B, Gerzanich V, Simard JM (2012). Hemorrhagic progression of a contusion after traumatic brain injury: a review. J Neurotrauma.

[55] Wang F, Hu S, Ding Y, Ju X, Wang L, Lu Q, Wu X. Neutrophil-to-lymphocyte ratio and 30-day mortality in patients with acute intracerebral hemorrhage. J Stroke Cerebrovasc Dis. 2015; 10.1016/j.jstrokecerebrovasdis.2015.09.013 26500171

[56] Zhao X, Sun G, Zhang H, Ting SM, Song S, Gonzales N, Aronowski J (2014). Polymorphonuclear neutrophil in brain parenchyma after experimental intracerebral hemorrhage. Transl Stroke Res.

[57] Rosell A, Cuadrado E, Ortega-Aznar A, Hernandez-Guillamon M, Lo EH, Montaner J (2008). MMP-9-positive neutrophil infiltration is associated to blood-brain barrier breakdown and basal lamina type IV collagen degradation during hemorrhagic transformation after human ischemic stroke. Stroke.

[58] Clark RS, Kochanek PM, Schwarz MA, Schiding JK, Turner DS, Chen M, Carlos TM, Watkins SC (1996). Inducible nitric oxide synthase expression in cerebrovascular smooth muscle and neutrophils after traumatic brain injury in immature rats. Pediatr Res.

[59] Johanson C, Stopa E, Baird A, Sharma H (2011). Traumatic brain injury and recovery mechanisms: peptide modulation of periventricular neurogenic regions by the choroid plexus-CSF nexus. J Neural Transm (Vienna).

[60] Baruch K, Kertser A, Porat Z, Schwartz M (2015). Cerebral nitric oxide represses choroid plexus NFkappaB-dependent gateway activity for leukocyte trafficking. EMBO J.

[61] Szmydynger-Chodobska J, Strazielle N, Zink BJ, Ghersi-Egea JF, Chodobski A (2009). The role of the choroid plexus in neutrophil invasion after traumatic brain injury. J Cereb Blood Flow Metab.

[62] Carlos TM, Clark RS, Franicola-Higgins D, Schiding JK, Kochanek PM (1997). Expression of endothelial adhesion molecules and recruitment of neutrophils after traumatic brain injury in rats. J Leukoc Biol.

[63] Steffen BJ, Breier G, Butcher EC, Schulz M, Engelhardt B (1996). ICAM-1, VCAM-1, and MAdCAM-1 are expressed on choroid plexus epithelium but not endothelium and mediate binding of lymphocytes in vitro. Am J Pathol.

[64] Zetterberg H, Smith DH, Blennow K (2013). Biomarkers of mild traumatic brain injury in cerebrospinal fluid and blood. Nat Rev Neurol.

[65] Baker AJ, Moulton RJ, MacMillan VH, Shedden PM (1993). Excitatory amino acids in cerebrospinal fluid following traumatic brain injury in humans. J Neurosurg.

[66] Kossmann T, Hans V, Imhof HG, Trentz O, Morganti-Kossmann MC (1996). Interleukin-6 released in human cerebrospinal fluid following traumatic brain injury may trigger nerve growth factor production in astrocytes. Brain Res.

[67] Gupta R, Palchaudhuri S, Chattopadhyay D (2013). Glutamate induces neutrophil cell migration by activating class I metabotropic glutamate receptors. Amino Acids.

[68] Yang T, Liu YW, Zhao L, Wang H, Yang N, Dai SS, He F (2017). Metabotropic glutamate receptor 5 deficiency inhibits neutrophil infiltration after traumatic brain injury in mice. Sci Rep.

[69] Sagiv JY, Michaeli J, Assi S, Mishalian I, Kisos H, Levy L, Damti P, Lumbroso D, Polyansky L, Sionov RV (2015). Phenotypic diversity and plasticity in circulating neutrophil subpopulations in cancer. Cell Rep.

[70] Chodobski A, Chung I, Kozniewska E, Ivanenko T, Chang W, Harrington JF, Duncan JA, Szmydynger-Chodobska J (2003). Early neutrophilic expression of vascular endothelial growth factor after traumatic brain injury. Neuroscience.

[71] Stins MF, Gilles F, Kim KS (1997). Selective expression of adhesion molecules on human brain microvascular endothelial cells. J Neuroimmunol.

[72] Engelhardt B, Conley FK, Butcher EC (1994). Cell adhesion molecules on vessels during inflammation in the mouse central nervous system. J Neuroimmunol.

[73] Ji K, Eu MY, Kang SH, Gwag BJ, Jou I, Joe EH (2008). Differential neutrophil infiltration contributes to regional differences in brain inflammation in the substantia nigra pars compacta and cortex. Glia.

[74] Obermeier B, Verma A, Ransohoff RM (2016). The blood-brain barrier. Handb Clin Neurol.

[75] von Wedel-Parlow M, Schrot S, Lemmen J, Treeratanapiboon L, Wegener J, Galla HJ (2011). Neutrophils cross the BBB primarily on transcellular pathways: an in vitro study. Brain Res.

[76] Soares HD, Hicks RR, Smith D, McIntosh TK (1995). Inflammatory leukocytic recruitment and diffuse neuronal degeneration are separate pathological processes resulting from traumatic brain injury. J Neurosci.

[77] Lu KT, Wang YW, Yang JT, Yang YL, Chen HI (2005). Effect of interleukin-1 on traumatic brain injury-induced damage to hippocampal neurons. J Neurotrauma.

[78] Nourshargh S, Alon R (2014). Leukocyte migration into inflamed tissues. Immunity.

[79] Zhang R, Chopp M, Li Y, Zaloga C, Jiang N, Jones M, Miyasaka M, Ward P (1994). Anti-ICAM-1 antibody reduces ischemic cell damage after transient middle cerebral artery occlusion in the rat. Neurology.

[80] Zhang RL, Chopp M, Jiang N, Tang WX, Prostak J, Manning AM, Anderson DC (1995). Anti–intercellular adhesion molecule–1 antibody reduces ischemic cell damage after transient but not permanent middle cerebral artery occlusion in the Wistar rat. Stroke.

[81] Arumugam TV, Salter JW, Chidlow JH, Ballantyne CM, Kevil CG, Granger DN (2004). Contributions of LFA-1 and Mac-1 to brain injury and microvascular dysfunction induced by transient middle cerebral artery occlusion. Am J Physiol Heart Circ Physiol.

[82] Whalen MJ, Carlos TM, Dixon CE, Robichaud P, Clark RS, Marion DW, Kochanek PM (2000). Reduced brain edema after traumatic brain injury in mice deficient in P-selectin and intercellular adhesion molecule-1. J Leukoc Biol.

[83] Semple BD, Bye N, Ziebell JM, Morganti-Kossmann MC (2010). Deficiency of the chemokine receptor CXCR2 attenuates neutrophil infiltration and cortical damage following closed head injury. Neurobiol Dis.

[84] Connolly ES, Winfree CJ, Springer TA, Naka Y, Liao H, Yan SD, Stern DM, Solomon RA, Gutierrez-Ramos JC, Pinsky DJ (1996). Cerebral protection in homozygous null ICAM-1 mice after middle cerebral artery occlusion. Role of neutrophil adhesion in the pathogenesis of stroke. J Clin Invest.

[85] Sousa LFC, Coelho FM, Rodrigues DH, Campos AC, Barcelos LS, Teixeira MM, Rachid MA, Teixeira AL (2013). Blockade of CXCR1/2 chemokine receptors protects against brain damage in ischemic stroke in mice. Clinics.

[86] Huang J, Li Y, Tang Y, Tang G, Yang G-Y, Wang Y (2013). CXCR4 antagonist AMD3100 protects blood–brain barrier integrity and reduces inflammatory response after focal ischemia in mice. Stroke.

[87] Bolton SJ, Anthony DC, Perry VH (1998). Loss of the tight junction proteins occludin and zonula occludens-1 from cerebral vascular endothelium during neutrophil-induced blood-brain barrier breakdown in vivo. Neuroscience.

[88] Allport JR, Ding H, Collins T, Gerritsen ME, Luscinskas FW (1997). Endothelial-dependent mechanisms regulate leukocyte transmigration: a process involving the proteasome and disruption of the vascular endothelial-cadherin complex at endothelial cell-to-cell junctions. J Exp Med.

[89] Hayashi T, Kaneko Y, Yu S, Bae E, Stahl CE, Kawase T, van Loveren H, Sanberg PR, Borlongan CV (2009). Quantitative analyses of matrix metalloproteinase activity after traumatic brain injury in adult rats. Brain Res.

[90] Grossetete M, Phelps J, Arko L, Yonas H, Rosenberg GA (2009). Elevation of matrix metalloproteinases 3 and 9 in cerebrospinal fluid and blood in patients with severe traumatic brain injury. Neurosurgery.

[91] Chen XR, Besson VC, Palmier B, Garcia Y, Plotkine M, Marchand-Leroux C (2007). Neurological recovery-promoting, anti-inflammatory, and anti-oxidative effects afforded by fenofibrate, a PPAR alpha agonist, in traumatic brain injury. J Neurotrauma.

[92] Mori T, Wang X, Aoki T, Lo EH (2002). Downregulation of matrix metalloproteinase-9 and attenuation of edema via inhibition of ERK mitogen activated protein kinase in traumatic brain injury. J Neurotrauma.

[93] Asahi M, Asahi K, Jung J-C, Del Zoppo GJ, Fini ME, Lo EH (2000). Role for matrix metalloproteinase 9 after focal cerebral ischemia: effects of gene knockout and enzyme inhibition with BB-94. J Cereb Blood Flow Metab.

[94] Gasche Y, Copin J-C, Sugawara T, Fujimura M, Chan PH (2001). Matrix metalloproteinase inhibition prevents oxidative stress-associated blood–brain barrier disruption after transient focal cerebral ischemia. J Cereb Blood Flow Metab.

[95] Hu Q, Chen C, Khatibi NH, Li L, Yang L, Wang K, Han J, Duan W, Zhang JH, Zhou C (2011). Lentivirus-mediated transfer of MMP-9 shRNA provides neuroprotection following focal ischemic brain injury in rats. Brain Res.

[96] Liu X, Sui B, Sun J (2017). Blood-brain barrier dysfunction induced by silica NPs in vitro and in vivo: involvement of oxidative stress and Rho-kinase/JNK signaling pathways. Biomaterials.

[97] Pun PB, Lu J, Moochhala S (2009). Involvement of ROS in BBB dysfunction. Free Radic Res.

[98] Chen G, Shi J, Hu Z, Hang C (2008). Inhibitory effect on cerebral inflammatory response following traumatic brain injury in rats: a potential neuroprotective mechanism of N-acetylcysteine. Mediat Inflamm.

[99] Wada K, Chatzipanteli K, Busto R, Dietrich WD (1998). Role of nitric oxide in traumatic brain injury in the rat. J Neurosurg.

[100] Saffarzadeh M, Juenemann C, Queisser MA, Lochnit G, Barreto G, Galuska SP, Lohmeyer J, Preissner KT (2012). Neutrophil extracellular traps directly induce epithelial and endothelial cell death: a predominant role of histones. PLoS One.

[101] Qureshi AI, Suarez JI (2000). Use of hypertonic saline solutions in treatment of cerebral edema and intracranial hypertension. Crit Care Med.

[102] Klatzo I (1987). Pathophysiological aspects of brain edema. Acta Neuropathol.

[103] Lu Q, Harrington EO, Newton J, Casserly B, Radin G, Warburton R, Zhou Y, Blackburn MR, Rounds S (2010). Adenosine protected against pulmonary edema through transporter- and receptor A2-mediated endothelial barrier enhancement. Am J Physiol Lung Cell Mol Physiol.

[104] Harris AK, Ergul A, Kozak A, Machado LS, Johnson MH, Fagan SC (2005). Effect of neutrophil depletion on gelatinase expression, edema formation and hemorrhagic transformation after focal ischemic stroke. BMC Neuroscience.

[105] Kim JE, Ryu HJ, Choi SY, Kang TC (2012). Tumor necrosis factor-alpha-mediated threonine 435 phosphorylation of p65 nuclear factor-kappaB subunit in endothelial cells induces vasogenic edema and neutrophil infiltration in the rat piriform cortex following status epilepticus. J Neuroinflammation.

[106] Liang D, Bhatta S, Gerzanich V, Simard JM (2007). Cytotoxic edema: mechanisms of pathological cell swelling. Neurosurg Focus.

[107] Suzuki Y, Matsumoto Y, Ikeda Y, Kondo K, Ohashi N, Umemura K (2002). SM-20220, a Na(+)/H(+) exchanger inhibitor: effects on ischemic brain damage through edema and neutrophil accumulation in a rat middle cerebral artery occlusion model. Brain Res.

[108] Osaki M, Sumimoto H, Takeshige K, Cragoe EJ, Hori Y, Minakami S (1989). Na+/H+ exchange modulates the production of leukotriene B4 by human neutrophils. Biochem J.

[109] Edens HA, Parkos CA (2003). Neutrophil transendothelial migration and alteration in vascular permeability: focus on neutrophil-derived azurocidin. Curr Opin Hematol.

[110] Takano T, Clish CB, Gronert K, Petasis N, Serhan CN (1998). Neutrophil-mediated changes in vascular permeability are inhibited by topical application of aspirin-triggered 15-epi-lipoxin A4 and novel lipoxin B4 stable analogues. J Clin Invest.

[111] Ikegame Y, Yamashita K, Hayashi S, Yoshimura S, Nakashima S, Iwama T (2010). Neutrophil elastase inhibitor prevents ischemic brain damage via reduction of vasogenic edema. Hypertens Res.

[112] Zheng H, Chen C, Zhang J, Hu Z (2016). Mechanism and therapy of brain edema after intracerebral hemorrhage. Cerebrovascular Diseases.

[113] Stirling DP, Liu S, Kubes P, Yong VW (2009). Depletion of Ly6G/Gr-1 leukocytes after spinal cord injury in mice alters wound healing and worsens neurological outcome. J Neurosci.

[114] Chang JJ, Youn TS, Benson D, Mattick H, Andrade N, Harper CR, Moore CB, Madden CJ, Diaz-Arrastia RR (2009). Physiologic and functional outcome correlates of brain tissue hypoxia in traumatic brain injury. Crit Care Med.

[115] Stiefel MF, Udoetuk JD, Spiotta AM, Gracias VH, Goldberg A, Maloney-Wilensky E, Bloom S, Le Roux PD (2006). Conventional neurocritical care and cerebral oxygenation after traumatic brain injury. J Neurosurg.

[116] Walmsley SR, Print C, Farahi N, Peyssonnaux C, Johnson RS, Cramer T, Sobolewski A, Condliffe AM, Cowburn AS, Johnson N, Chilvers ER (2005). Hypoxia-induced neutrophil survival is mediated by HIF-1alpha-dependent NF-kappaB activity. J Exp Med.

[117] Reiss M, Roos D (1978). Differences in oxygen metabolism of phagocytosing monocytes and neutrophils. J Clin Invest.

[118] Segal AW, Coade SB (1978). Kinetics of oxygen consumption by phagocytosing human neutrophils. Biochem Biophys Res Commun.

[119] Lewen A, Hillered L (1998). Involvement of reactive oxygen species in membrane phospholipid breakdown and energy perturbation after traumatic brain injury in the rat. J Neurotrauma.

[120] Schluesener H, Meyermann R (1995). Neutrophilic defensins penetrate the blood-brain barrier. J Neurosci Res.

[121] De Y, Chen Q, Schmidt AP, Anderson GM, Wang JM, Wooters J, Oppenheim JJ, Chertov O (2000). LL-37, the neutrophil granule- and epithelial cell-derived cathelicidin, utilizes formyl peptide receptor-like 1 (FPRL1) as a receptor to chemoattract human peripheral blood neutrophils, monocytes, and T cells. J Exp Med.

[122] Taylor PR, Roy S, Leal SM, Sun Y, Howell SJ, Cobb BA, Li X, Pearlman E (2014). Activation of neutrophils by autocrine IL-17A–IL-17RC interactions during fungal infection is regulated by IL-6, IL-23, RORγt and dectin-2. Nat Immunol.

[123] Beray-Berthat V, Palmier B, Plotkine M, Margaill I (2003). Neutrophils do not contribute to infarction, oxidative stress, and NO synthase activity in severe brain ischemia. Exp Neurol.

[124] Rodrigo J, Fernandez A, Serrano J, Peinado M, Martinez A (2005). The role of free radicals in cerebral hypoxia and ischemia. Free Radic Biol Med.

[125] Bramlett HM, Dietrich WD, Green EJ (1999). Secondary hypoxia following moderate fluid percussion brain injury in rats exacerbates sensorimotor and cognitive deficits. J Neurotrauma.

[126] Kumar A, Loane DJ (2012). Neuroinflammation after traumatic brain injury: opportunities for therapeutic intervention. Brain Behav Immun.

[127] Lampron A, Elali A, Rivest S (2013). Innate immunity in the CNS: redefining the relationship between the CNS and its environment. Neuron.

[128] Moxon-Emre I, Schlichter LC (2011). Neutrophil depletion reduces blood-brain barrier breakdown, axon injury, and inflammation after intracerebral hemorrhage. J Neuropathol Exp Neurol.

[129] Zhou H, Lapointe BM, Clark SR, Zbytnuik L, Kubes P (2006). A requirement for microglial TLR4 in leukocyte recruitment into brain in response to lipopolysaccharide. J Immunol.

[130] Loane DJ, Kumar A (2016). Microglia in the TBI brain: the good, the bad, and the dysregulated. Exp Neurol.

[131] Qin L, Li G, Qian X, Liu Y, Wu X, Liu B, Hong JS, Block ML (2005). Interactive role of the toll-like receptor 4 and reactive oxygen species in LPS-induced microglia activation. Glia.

[132] Jang E, Lee S, Kim JH, Kim JH, Seo JW, Lee WH, Mori K, Nakao K, Suk K (2013). Secreted protein lipocalin-2 promotes microglial M1 polarization. FASEB J.

[133] McPherson CA, Merrick BA, Harry GJ (2014). In vivo molecular markers for pro-inflammatory cytokine M1 stage and resident microglia in trimethyltin-induced hippocampal injury. Neurotox Res.

[134] Imai F, Suzuki H, Oda J, Ninomiya T, Ono K, Sano H, Sawada M (2007). Neuroprotective effect of exogenous microglia in global brain ischemia. J Cereb Blood Flow Metab.

[135] Lalancette-Hébert M, Gowing G, Simard A, Weng YC, Kriz J (2007). Selective ablation of proliferating microglial cells exacerbates ischemic injury in the brain. J Neurosci.

[136] Neumann J, Sauerzweig S, Ronicke R, Gunzer F, Dinkel K, Ullrich O, Gunzer M, Reymann KG (2008). Microglia cells protect neurons by direct engulfment of invading neutrophil granulocytes: a new mechanism of CNS immune privilege. J Neurosci.

[137] Seo JH, Miyamoto N, Hayakawa K, Pham L-DD, Maki T, Ayata C, Kim K-W, Lo EH, Arai K (2013). Oligodendrocyte precursors induce early blood-brain barrier opening after white matter injury. J Clin Invest.

[138] Tani M, Fuentes ME, Peterson JW, Trapp BD, Durham SK, Loy JK, Bravo R, Ransohoff RM, Lira SA (1996). Neutrophil infiltration, glial reaction, and neurological disease in transgenic mice expressing the chemokine N51/KC in oligodendrocytes. J Clin Investig.

[139] Chen S-C, Leach MW, Chen Y, Cai X-Y, Sullivan L, Wiekowski M, Dovey-Hartman B, Zlotnik A, Lira SA (2002). Central nervous system inflammation and neurological disease in transgenic mice expressing the CC chemokine CCL21 in oligodendrocytes. J Immunol.

[140] Liu L, Belkadi A, Darnall L, Hu T, Drescher C, Cotleur AC, Padovani-Claudio D, He T, Choi K, Lane TE (2010). CXCR2+ neutrophils play an essential role in cuprizone-induced demyelination: relevance to multiple sclerosis. Nat Neurosci.

[141] Liu Z, Chopp M. Astrocytes, therapeutic targets for neuroprotection and neurorestoration in ischemic stroke. Prog Neurobiol. 2015; 10.1016/j.pneurobio.2015.09.008 PMC4826643 26455456

[142] Lee SC, Liu W, Dickson DW, Brosnan CF, Berman JW (1993). Cytokine production by human fetal microglia and astrocytes. Differential induction by lipopolysaccharide and IL-1 beta. J Immunol.

[143] Gottschall PE, Yu X (1995). Cytokines regulate gelatinase A and B (matrix metalloproteinase 2 and 9) activity in cultured rat astrocytes. J Neurochem.

[144] Kalish H, Phillips TM (2009). Application of immunoaffinity capillary electrophoresis to the measurements of secreted cytokines by cultured astrocytes. J Sep Sci.

[145] Pineau I, Sun L, Bastien D, Lacroix S (2010). Astrocytes initiate inflammation in the injured mouse spinal cord by promoting the entry of neutrophils and inflammatory monocytes in an IL-1 receptor/MyD88-dependent fashion. Brain Behav Immun.

[146] Lu W, Maheshwari A, Misiuta I, Fox SE, Chen N, Zigova T, Christensen RD, Calhoun DA (2005). Neutrophil-specific chemokines are produced by astrocytic cells but not by neuronal cells. Brain Res Dev Brain Res.

[147] Savarin C, Stohlman SA, Rietsch AM, Butchi N, Ransohoff RM, Bergmann CC (2011). MMP9 deficiency does not decrease blood-brain barrier disruption, but increases astrocyte MMP3 expression during viral encephalomyelitis. Glia.

[148] Fang J, Han D, Hong J, Tan Q, Tian Y (2012). The chemokine, macrophage inflammatory protein-2gamma, reduces the expression of glutamate transporter-1 on astrocytes and increases neuronal sensitivity to glutamate excitotoxicity. J Neuroinflammation.

[149] Eugenin EA, D'Aversa TG, Lopez L, Calderon TM, Berman JW (2003). MCP-1 (CCL2) protects human neurons and astrocytes from NMDA or HIV-tat-induced apoptosis. J Neurochem.

[150] Hu S, Sheng WS, Ehrlich LC, Peterson PK, Chao CC (2000). Cytokine effects on glutamate uptake by human astrocytes. Neuroimmunomodulation.

[151] Voskuhl RR, Peterson RS, Song B, Ao Y, Morales LBJ, Tiwari-Woodruff S, Sofroniew MV (2009). Reactive astrocytes form scar-like perivascular barriers to leukocytes during adaptive immune inflammation of the CNS. J Neurosci.

[152] Xie L, Poteet EC, Li W, Scott AE, Liu R, Wen Y, Ghorpade A, Simpkins JW, Yang SH (2010). Modulation of polymorphonuclear neutrophil functions by astrocytes. J Neuroinflammation.

[153] Hooshmand MJ, Nguyen HX (2017). Neutrophils induce astroglial differentiation and migration of human neural stem cells via C1q and C3a synthesis.

[154] Johnson VE, Stewart JE, Begbie FD, Trojanowski JQ, Smith DH, Stewart W (2013). Inflammation and white matter degeneration persist for years after a single traumatic brain injury. Brain.

[155] Plassman BL, Havlik RJ, Steffens DC, Helms MJ, Newman TN, Drosdick D, Phillips C, Gau BA, Welsh-Bohmer KA, Burke JR (2000). Documented head injury in early adulthood and risk of Alzheimer’s disease and other dementias. Neurology.

[156] Ryu JK, Tran KC, McLarnon JG (2007). Depletion of neutrophils reduces neuronal degeneration and inflammatory responses induced by quinolinic acid in vivo. Glia.

[157] Zenaro E, Pietronigro E, Della Bianca V, Piacentino G, Marongiu L, Budui S, Turano E, Rossi B, Angiari S, Dusi S (2015). Neutrophils promote Alzheimer’s disease-like pathology and cognitive decline via LFA-1 integrin. Nat Med.

[158] Baik SH, Cha MY, Hyun YM, Cho H, Hamza B, Kim DK, Han SH, Choi H, Kim KH, Moon M (2014). Migration of neutrophils targeting amyloid plaques in Alzheimer’s disease mouse model. Neurobiol Aging.

[159] Woodruff TM, Crane JW, Proctor LM, Buller KM, Shek AB, De Vos K, Pollitt S, Williams HM, Shiels IA, Monk PN (2006). Therapeutic activity of C5a receptor antagonists in a rat model of neurodegeneration. FASEB J.

[160] Vitte J, Michel BF, Bongrand P, Gastaut JL (2004). Oxidative stress level in circulating neutrophils is linked to neurodegenerative diseases. J Clin Immunol.

[161] Simi A, Tsakiri N, Wang P, Rothwell N. Interleukin-1 and inflammatory neurodegeneration: Portland Press Limited; 2007. 10.1042/BST0351122 17956293

[162] Shaftel SS, Carlson TJ, Olschowka JA, Kyrkanides S, Matousek SB, O'Banion MK (2007). Chronic interleukin-1β expression in mouse brain leads to leukocyte infiltration and neutrophil-independent blood–brain barrier permeability without overt neurodegeneration. J Neurosci.

[163] Kumar DKV, Choi SH, Washicosky KJ, Eimer WA, Tucker S, Ghofrani J, Lefkowitz A, McColl G, Goldstein LE, Tanzi RE (2016). Amyloid-β peptide protects against microbial infection in mouse and worm models of Alzheimer’s disease. Sci Transl Med.

[164] Dinkel K, Dhabhar FS, Sapolsky RM (2004). Neurotoxic effects of polymorphonuclear granulocytes on hippocampal primary cultures. Proc Natl Acad Sci U S A.

[165] Tang J, Liu J, Zhou C, Alexander JS, Nanda A, Granger DN, Zhang JH (2004). Mmp-9 deficiency enhances collagenase-induced intracerebral hemorrhage and brain injury in mutant mice. J Cereb Blood Flow Metab.

[166] Semple BD, Trivedi A, Gimlin K, Noble-Haeusslein LJ. Neutrophil elastase mediates acute pathogenesis and is a determinant of long-term behavioral recovery after traumatic injury to the immature brain. Neurobiol Dis. 2014; 10.1016/j.nbd.2014.12.003 PMC4323771 25497734

[167] Chung-ha OD, Kim K-Y, Bushong EA, Mills EA, Boassa D, Shih T, Kinebuchi M, Phan S, Zhou Y, Bihlmeyer NA (2014). Transcellular degradation of axonal mitochondria. Proc Natl Acad Sci.

[168] Hayakawa K, Esposito E, Wang X, Terasaki Y, Liu Y, Xing C, Ji X, Lo EH (2016). Transfer of mitochondria from astrocytes to neurons after stroke. Nature.

[169] Leow-Dyke S, Allen C, Denes A, Nilsson O, Maysami S, Bowie AG, Rothwell NJ, Pinteaux E (2012). Neuronal Toll-like receptor 4 signaling induces brain endothelial activation and neutrophil transmigration in vitro. J Neuroinflammation.

[170] Raposo C, Schwartz M (2014). Glial scar and immune cell involvement in tissue remodeling and repair following acute CNS injuries. Glia.

[171] Roth TL, Nayak D, Atanasijevic T, Koretsky AP, Latour LL, McGavern DB (2014). Transcranial amelioration of inflammation and cell death after brain injury. Nature.

[172] Kurimoto T, Yin Y, Habboub G, Gilbert HY, Li Y, Nakao S, Hafezi-Moghadam A, Benowitz LI (2013). Neutrophils express oncomodulin and promote optic nerve regeneration. J Neurosci.

[173] Grotendorst GR, Smale G, Pencev D (1989). Production of transforming growth factor beta by human peripheral blood monocytes and neutrophils. J Cell Physiol.

[174] Lindholm D, Castren E, Kiefer R, Zafra F, Thoenen H (1992). Transforming growth factor-beta 1 in the rat brain: increase after injury and inhibition of astrocyte proliferation. J Cell Biol.

[175] Schneider A, Krüger C, Steigleder T, Weber D, Pitzer C, Laage R, Aronowski J, Maurer MH, Gassler N, Mier W (2005). The hematopoietic factor G-CSF is a neuronal ligand that counteracts programmed cell death and drives neurogenesis. J Clin Invest.

[176] Honda S, Kagoshima M, Wanaka A, Tohyama M, Matsumoto K, Nakamura T (1995). Localization and functional coupling of HGF and c-Met/HGF receptor in rat brain: implication as neurotrophic factor. Brain Res Mol Brain Res.

[177] Kossmann T, Stahel PF, Lenzlinger PM, Redl H, Dubs RW, Trentz O, Schlag G, Morganti-Kossmann MC (1997). Interleukin-8 released into the cerebrospinal fluid after brain injury is associated with blood-brain barrier dysfunction and nerve growth factor production. J Cereb Blood Flow Metab.

[178] Pérez-Navarro E, Canudas AM, Åkerud P, Alberch J, Arenas E (2000). Brain-derived neurotrophic factor, neurotrophin-3, and neurotrophin-4/5 prevent the death of striatal projection neurons in a rodent model of Huntington’s disease. J Neurochem.

[179] Owada K, Sanjo N, Kobayashi T, Mizusawa H, Muramatsu H, Muramatsu T, Michikawa M (1999). Midkine inhibits caspase-dependent apoptosis via the activation of mitogen-activated protein kinase and phosphatidylinositol 3-kinase in cultured neurons. J Neurochem.

[180] Ancelin M, Chollet-Martin S, Hervé MA, Legrand C, El Benna J, Perrot-Applanat M (2004). Vascular endothelial growth factor VEGF189 induces human neutrophil chemotaxis in extravascular tissue via an autocrine amplification mechanism. Lab Investig.

[181] Belperio JA, Keane MP, Arenberg DA, Addison CL, Ehlert JE, Burdick MD, Strieter RM (2000). CXC chemokines in angiogenesis. J Leukoc Biol.

[182] Salmeron K, Aihara T, Redondo-Castro E, Pinteaux E, Bix G (2016). IL-1alpha induces angiogenesis in brain endothelial cells in vitro: implications for brain angiogenesis after acute injury. J Neurochem.

[183] Gidday JM, Gasche YG, Copin JC, Shah AR, Perez RS, Shapiro SD, Chan PH, Park TS (2005). Leukocyte-derived matrix metalloproteinase-9 mediates blood-brain barrier breakdown and is proinflammatory after transient focal cerebral ischemia. Am J Physiol Heart Circ Physiol.

[184] Raffetto JD, Khalil RA (2008). Matrix metalloproteinases and their inhibitors in vascular remodeling and vascular disease. Biochem Pharmacol.

[185] Zhang ZG, Zhang L, Jiang Q, Zhang R, Davies K, Powers C, Bruggen N, Chopp M (2000). VEGF enhances angiogenesis and promotes blood-brain barrier leakage in the ischemic brain. J Clin Invest.

[186] Gasser O, Schifferli JA (2004). Activated polymorphonuclear neutrophils disseminate anti-inflammatory microparticles by ectocytosis. Blood.

[187] Musiani P, Allione A, Modica A, Lollini PL, Giovarelli M, Cavallo F, Belardelli F, Forni G, Modesti A (1996). Role of neutrophils and lymphocytes in inhibition of a mouse mammary adenocarcinoma engineered to release IL-2, IL-4, IL-7, IL-10, IFN-alpha, IFN-gamma, and TNF-alpha. Lab Investig.

[188] Brandes ME, Mai UE, Ohura K, Wahl SM (1991). Type I transforming growth factor-beta receptors on neutrophils mediate chemotaxis to transforming growth factor-beta. J Immunol.

[189] Matsuo Y, Onodera H, Shiga Y, Nakamura M, Ninomiya M, Kihara T, Kogure K (1994). Correlation between myeloperoxidase-quantified neutrophil accumulation and ischemic brain injury in the rat. Effects of neutrophil depletion. Stroke.

[190] Carr KD, Sieve AN, Indramohan M, Break TJ, Lee S, Berg RE (2011). Specific depletion reveals a novel role for neutrophil-mediated protection in the liver during Listeria monocytogenes infection. Eur J Immunol.

[191] Navarini AA, Lang KS, Verschoor A, Recher M, Zinkernagel AS, Nizet V, Odermatt B, Hengartner H, Zinkernagel RM (2009). Innate immune-induced depletion of bone marrow neutrophils aggravates systemic bacterial infections. Proc Natl Acad Sci.

[192] Lewis SM, Khan N, Beale R, Treacher DF, Brown KA (2013). Depletion of blood neutrophils from patients with sepsis: treatment for the future?. Int Immunopharmacol.

[193] Dela Pena I, Sanberg PR, Acosta S, Tajiri N, Lin SZ, Borlongan CV (2014). Stem cells and G-CSF for treating neuroinflammation in traumatic brain injury: aging as a comorbidity factor. J Neurosurg Sci.

[194] Fridlender ZG, Sun J, Kim S, Kapoor V, Cheng G, Ling L, Worthen GS, Albelda SM (2009). Polarization of tumor-associated neutrophil phenotype by TGF-beta: “N1” versus “N2” TAN. Cancer Cell.

[195] Tsuda Y, Takahashi H, Kobayashi M, Hanafusa T, Herndon DN, Suzuki F (2004). Three different neutrophil subsets exhibited in mice with different susceptibilities to infection by methicillin-resistant Staphylococcus aureus. Immunity.

[196] Cuartero MI, Ballesteros I, Moraga A, Nombela F, Vivancos J, Hamilton JA, Corbí ÁL, Lizasoain I, Moro MA (2013). N2 neutrophils, novel players in brain inflammation after stroke. Stroke.

[197] Tak T, Tesselaar K, Pillay J, Borghans JA, Koenderman L (2013). What's your age again? Determination of human neutrophil half-lives revisited. J Leukoc Biol.

[198] Pillay J, den Braber I, Vrisekoop N, Kwast LM, de Boer RJ, Borghans JA, Tesselaar K, Koenderman L (2010). In vivo labeling with 2H2O reveals a human neutrophil lifespan of 5.4 days. Blood.

[199] Pithon-Curi TC, Schumacher RI, Freitas JJ, Lagranha C, Newsholme P, Palanch AC, Doi SQ, Curi R (2003). Glutamine delays spontaneous apoptosis in neutrophils. Am J Phys Cell Phys.

[200] Vaughan KR, Stokes L, Prince LR, Marriott HM, Meis S, Kassack MU, Bingle CD, Sabroe I, Surprenant A, Whyte MK (2007). Inhibition of neutrophil apoptosis by ATP is mediated by the P2Y11 receptor. J Immunol.

[201] Liu YW, Yang T, Zhao L, Ni Z, Yang N, He F, Dai SS (2016). Activation of Adenosine 2A receptor inhibits neutrophil apoptosis in an autophagy-dependent manner in mice with systemic inflammatory response syndrome. Sci Rep.

[202] Pillay J, Kamp VM, van Hoffen E, Visser T, Tak T, Lammers JW, Ulfman LH, Leenen LP, Pickkers P, Koenderman L (2012). A subset of neutrophils in human systemic inflammation inhibits T cell responses through Mac-1. J Clin Invest.

[203] Gabrilovich DI, Nagaraj S (2009). Myeloid-derived-suppressor cells as regulators of the immune system. Nat Rev Immunol.

[204] Proebstl D, Voisin MB, Woodfin A, Whiteford J, D'Acquisto F, Jones GE, Rowe D, Nourshargh S (2012). Pericytes support neutrophil subendothelial cell crawling and breaching of venular walls in vivo. J Exp Med.

[205] Armulik A, Genové G, Mäe M, Nisancioglu MH, Wallgard E, Niaudet C, He L, Norlin J, Lindblom P, Strittmatter K (2010). Pericytes regulate the blood-brain barrier. Nature.

[206] Dai SS, Zhou YG, Li W, An JH, Li P, Yang N, Chen XY, Xiong RP, Liu P, Zhao Y (2010). Local glutamate level dictates adenosine A2A receptor regulation of neuroinflammation and traumatic brain injury. J Neurosci.

[207] Chakravarti A, Rusu D, Flamand N, Borgeat P, Poubelle PE (2009). Reprogramming of a subpopulation of human blood neutrophils by prolonged exposure to cytokines. Lab Investig.

[208] Robertson AL, Holmes GR, Bojarczuk AN, Burgon J, Loynes CA, Chimen M, Sawtell AK, Hamza B, Willson J, Walmsley SR (2014). A zebrafish compound screen reveals modulation of neutrophil reverse migration as an anti-inflammatory mechanism. Sci Transl Med.

[209] Colom B, Bodkin JV, Beyrau M, Woodfin A, Ody C, Rourke C, Chavakis T, Brohi K, Imhof BA, Nourshargh S (2015). Leukotriene B 4-neutrophil elastase axis drives neutrophil reverse transendothelial cell migration in vivo. Immunity.

[210] Brown K, Brain S, Pearson J, Edgeworth J, Lewis S, Treacher D (2006). Neutrophils in development of multiple organ failure in sepsis. Lancet.

[211] Dai SS, Wang H, Yang N, An JH, Li W, Ning YL, Zhu PF, Chen JF, Zhou YG (2013). Plasma glutamate-modulated interaction of A2AR and mGluR5 on BMDCs aggravates traumatic brain injury-induced acute lung injury. J Exp Med.

[212] Luheshi NM, Kovács KJ, Lopez-Castejon G, Brough D, Denes A (2011). Interleukin-1α expression precedes IL-1β after ischemic brain injury and is localised to areas of focal neuronal loss and penumbral tissues. J Neuroinflammation.

[213] Nishihara T, Ochi M, Sugimoto K, Takahashi H, Yano H, Kumon Y, Ohnishi T, Tanaka J (2011). Subcutaneous injection containing IL-3 and GM-CSF ameliorates stab wound-induced brain injury in rats. Exp Neurol.

[214] Liu X, Liu J, Zhao S, Zhang H, Cai W, Cai M, Ji X, Leak RK, Gao Y, Chen J, Hu X (2016). Interleukin-4 is essential for microglia/macrophage M2 polarization and long-term recovery after cerebral ischemia. Stroke.

[215] Arand M, Melzner H, Kinzl L, Bruckner UB, Gebhard F (2001). Early inflammatory mediator response following isolated traumatic brain injury and other major trauma in humans. Langenbeck's Arch Surg.

[216] Moors M, Vudattu NK, Abel J, Kramer U, Rane L, Ulfig N, Ceccatelli S, Seyfert-Margolies V, Fritsche E, Maeurer MJ (2010). Interleukin-7 (IL-7) and IL-7 splice variants affect differentiation of human neural progenitor cells. Genes Immun.

[217] Mesples B, Fontaine RH, Lelievre V, Launay JM, Gressens P (2005). Neuronal TGF-beta1 mediates IL-9/mast cell interaction and exacerbates excitotoxicity in newborn mice. Neurobiol Dis.

[218] Schwab JM, Nguyen TD, Meyermann R, Schluesener HJ (2001). Human focal cerebral infarctions induce differential lesional interleukin-16 (IL-16) expression confined to infiltrating granulocytes, CD8+ T-lymphocytes and activated microglia/macrophages. J Neuroimmunol.

[219] Gelderblom M, Weymar A, Bernreuther C, Velden J, Arunachalam P, Steinbach K, Orthey E, Arumugam TV, Leypoldt F, Simova O (2012). Neutralization of the IL-17 axis diminishes neutrophil invasion and protects from ischemic stroke. Blood.

[220] Yatsiv I, Morganti-Kossmann MC, Perez D, Dinarello CA, Novick D, Rubinstein M, Otto VI, Rancan M, Kossmann T, Redaelli CA (2002). Elevated intracranial IL-18 in humans and mice after traumatic brain injury and evidence of neuroprotective effects of IL-18-binding protein after experimental closed head injury. J Cereb Blood Flow Metab.

[221] Hedtjarn M, Leverin AL, Eriksson K, Blomgren K, Mallard C, Hagberg H (2002). Interleukin-18 involvement in hypoxic-ischemic brain injury. J Neurosci.

[222] Shichita T, Sugiyama Y, Ooboshi H, Sugimori H, Nakagawa R, Takada I, Iwaki T, Okada Y, Iida M, Cua DJ (2009). Pivotal role of cerebral interleukin-17-producing gammadeltaT cells in the delayed phase of ischemic brain injury. Nat Med.

[223] Omatsu T, Cepinskas G, Clarson C, Patterson EK, Alharfi IM, Summers K, Couraud PO, Romero IA, Weksler B, Fraser DD (2014). CXCL1/CXCL8 (GROalpha/IL-8) in human diabetic ketoacidosis plasma facilitates leukocyte recruitment to cerebrovascular endothelium in vitro. Am J Physiol Endocrinol Metab.

[224] Rhodes JK, Sharkey J, Andrews PJ (2009). The temporal expression, cellular localization, and inhibition of the chemokines MIP-2 and MCP-1 after traumatic brain injury in the rat. J Neurotrauma.

[225] Gleissner CA, Shaked I, Little KM, Ley K (2010). CXC chemokine ligand 4 induces a unique transcriptome in monocyte-derived macrophages. J Immunol.

[226] Gouwy M, Ruytinx P, Radice E, Claudi F, Van Raemdonck K, Bonecchi R, Locati M, Struyf S (2016). CXCL4 and CXCL4L1 differentially affect monocyte survival and dendritic cell differentiation and phagocytosis. PLoS One.

[227] Wang LY, Tu YF, Lin YC, Huang CC (2016). CXCL5 signaling is a shared pathway of neuroinflammation and blood–brain barrier injury contributing to white matter injury in the immature brain. J Neuroinflammation.

[228] Semple BD, Kossmann T, Morganti-Kossmann MC (2010). Role of chemokines in CNS health and pathology: a focus on the CCL2/CCR2 and CXCL8/CXCR2 networks. J Cereb Blood Flow Metab.

[229] Ren G, Zhang L, Zhao X, Xu G, Zhang Y, Roberts AI, Zhao RC, Shi Y (2008). Mesenchymal stem cell-mediated immunosuppression occurs via concerted action of chemokines and nitric oxide. Cell Stem Cell.

[230] Fahlenkamp AV, Coburn M, Czaplik M, Ryang Y-M, Kipp M, Rossaint R, Beyer C (2011). Expression analysis of the early chemokine response 4 h after in vitro traumatic brain injury. Inflamm Res.

[231] Israelsson C, Bengtsson H, Kylberg A, Kullander K, Lewen A, Hillered L, Ebendal T (2008). Distinct cellular patterns of upregulated chemokine expression supporting a prominent inflammatory role in traumatic brain injury. J Neurotrauma.

[232] Omari KM, Chui R, Dorovini-Zis K (2004). Induction of beta-chemokine secretion by human brain microvessel endothelial cells via CD40/CD40L interactions. J Neuroimmunol.

[233] Israelsson C, Kylberg A, Bengtsson H, Hillered L, Ebendal T (2014). Interacting chemokine signals regulate dendritic cells in acute brain injury. PLoS One.

[234] Mennicken F, Maki R, de Souza EB, Quirion R (1999). Chemokines and chemokine receptors in the CNS: a possible role in neuroinflammation and patterning. Trends Pharmacol Sci.

[235] Sheibani N, Grabowski EF, Schoenfeld DA, Whalen MJ (2004). Effect of granulocyte colony-stimulating factor on functional and histopathologic outcome after traumatic brain injury in mice*. Crit Care Med.

[236] Santiago E, Mora L, Bautista M, Montesinos JJ, Martinez I, Ramos G, Zambrano IR, Manrique B, Weiss-Steider B (2001). Granulocyte colony-stimulating factor induces neutrophils to secrete macrophage colony-stimulating factor. Cytokine.

[237] Imai Y, Kohsaka S (2002). Intracellular signaling in M-CSF-induced microglia activation: role of Iba1. Glia.

[238] Guerra-Crespo M, Gleason D, Sistos A, Toosky T, Solaroglu I, Zhang JH, Bryant PJ, Fallon JH (2009). Transforming growth factor-alpha induces neurogenesis and behavioral improvement in a chronic stroke model. Neuroscience.

[239] Ayari B, El Hachimi KH, Yanicostas C, Landoulsi A, Soussi-Yanicostas N (2010). Prokineticin 2 expression is associated with neural repair of injured adult zebrafish telencephalon. J Neurotrauma.

[240] Cheng MY, Lee AG, Culbertson C, Sun G, Talati RK, Manley NC, Li X, Zhao H, Lyons DM, Zhou Q-Y (2012). Prokineticin 2 is an endangering mediator of cerebral ischemic injury. Proc Natl Acad Sci.

[241] Nguyen HX, O'Barr TJ, Anderson AJ (2007). Polymorphonuclear leukocytes promote neurotoxicity through release of matrix metalloproteinases, reactive oxygen species, and TNF-alpha. J Neurochem.

[242] Pliyev BK, Menshikov M (2012). Differential effects of the autophagy inhibitors 3-methyladenine and chloroquine on spontaneous and TNF-alpha-induced neutrophil apoptosis. Apoptosis.

[243] Wu A, Ying Z, Gomez-Pinilla F (2004). Dietary omega-3 fatty acids normalize BDNF levels, reduce oxidative damage, and counteract learning disability after traumatic brain injury in rats. J Neurotrauma.

[244] Wu H, Lu D, Jiang H, Xiong Y, Qu C, Li B, Mahmood A, Zhou D, Chopp M (2008). Simvastatin-mediated upregulation of VEGF and BDNF, activation of the PI3K/Akt pathway, and increase of neurogenesis are associated with therapeutic improvement after traumatic brain injury. J Neurotrauma.

[245] Muramatsu H, Shirahama H, Yonezawa S, Maruta H, Muramatsu T (1993). Midkine, a retinoic acid-inducible growth/differentiation factor: immunochemical evidence for the function and distribution. Dev Biol.

[246] Chen S-H, Benveniste EN (2004). Oncostatin M: a pleiotropic cytokine in the central nervous system. Cytokine Growth Factor Rev.

[247] Weiss TW, Samson AL, Niego B, Daniel PB, Medcalf RL (2006). Oncostatin M is a neuroprotective cytokine that inhibits excitotoxic injury in vitro and in vivo. FASEB J.

[248] Yeo JE, Kang SK (1772). Selenium effectively inhibits ROS-mediated apoptotic neural precursor cell death in vitro and in vivo in traumatic brain injury. Biochim Biophys Acta.

[249] Steiner J, Rafols D, Park HK, Katar MS, Rafols JA, Petrov T (2004). Attenuation of iNOS mRNA exacerbates hypoperfusion and upregulates endothelin-1 expression in hippocampus and cortex after brain trauma. Nitric Oxide.

[250] Bayir H, Kagan VE, Borisenko GG, Tyurina YY, Janesko KL, Vagni VA, Billiar TR, Williams DL, Kochanek PM (2005). Enhanced oxidative stress in iNOS-deficient mice after traumatic brain injury: support for a neuroprotective role of iNOS. J Cereb Blood Flow Metab.

[251] Wang X, Jung J, Asahi M, Chwang W, Russo L, Moskowitz MA, Dixon CE, Fini ME, Lo EH (2000). Effects of matrix metalloproteinase-9 gene knock-out on morphological and motor outcomes after traumatic brain injury. J Neurosci.

[252] Luo CL, Chen XP, Yang R, Sun YX, Li QQ, Bao HJ, Cao QQ, Ni H, Qin ZH, Tao LY (2010). Cathepsin B contributes to traumatic brain injury-induced cell death through a mitochondria-mediated apoptotic pathway. J Neurosci Res.

[253] Elssner A, Duncan M, Gavrilin M, Wewers MD (2004). A novel P2X7 receptor activator, the human cathelicidin-derived peptide LL37, induces IL-1 beta processing and release. J Immunol.

[254] Shimakura A, Kamanaka Y, Ikeda Y, Kondo K, Suzuki Y, Umemura K (2000). Neutrophil elastase inhibition reduces cerebral ischemic damage in the middle cerebral artery occlusion. Brain Res.

[255] Kenne E, Erlandsson A, Lindbom L, Hillered L, Clausen F (2012). Neutrophil depletion reduces edema formation and tissue loss following traumatic brain injury in mice. J Neuroinflammation.

[256] Heard SO, Fink MP, Gamelli RL, Solomkin JS, Joshi M, Trask AL, Fabian TC, Hudson LD, Gerold KB, Logan ED (1998). Effect of prophylactic administration of recombinant human granulocyte colony-stimulating factor (filgrastim) on the frequency of nosocomial infections in patients with acute traumatic brain injury or cerebral hemorrhage. Crit Care Med.

[257] Jones NC, Constantin D, Prior MJ, Morris PG, Marsden CA, Murphy S (2005). The neuroprotective effect of progesterone after traumatic brain injury in male mice is independent of both the inflammatory response and growth factor expression. Eur J Neurosci.

[258] Wang Y-C, Wang P-F, Fang H, Chen J, Xiong X-Y, Yang Q-W (2013). Toll-like receptor 4 antagonist attenuates intracerebral hemorrhage–induced brain injury. Stroke.

[259] Neumann J, Riek-Burchardt M, Herz J, Doeppner TR, Konig R, Hutten H, Etemire E, Mann L, Klingberg A, Fischer T, et al. Very-late-antigen-4 (VLA-4)-mediated brain invasion by neutrophils leads to interactions with microglia, increased ischemic injury and impaired behavior in experimental stroke. Acta Neuropathol. 2014; 10.1007/s00401-014-1355-2 25391494

[260] Becker K, Kindrick D, Relton J, Harlan J, Winn R (2001). Antibody to the α4 integrin decreases infarct size in transient focal cerebral ischemia in rats. Stroke.

[261] Bao F, Omana V, Brown A, Weaver LC (2012). The systemic inflammatory response after spinal cord injury in the rat is decreased by α4β1 integrin blockade. J Neurotrauma.

[262] Dekosky ST, Styren SD, O'Malley ME, Goss JR, Kochanek P, Marion D, Evans CH, Robbins PD (1996). Interleukm-1 receptor antagonist suppresses neurotrophin response in injured rat brain. Ann Neurol.

[263] Toulmond S, Rothwell N (1995). Interleukin-1 receptor antagonist inhibits neuronal damage caused by fluid percussion injury in the rat. Brain Res.

[264] de Luca A, Smeekens SP, Casagrande A, Iannitti R, Conway KL, Gresnigt MS, Begun J, Plantinga TS, Joosten LA, van der Meer JW (2014). IL-1 receptor blockade restores autophagy and reduces inflammation in chronic granulomatous disease in mice and in humans. Proc Natl Acad Sci U S A.

[265] Villa P, Triulzi S, Cavalieri B, Di Bitondo R, Bertini R, Barbera S, Bigini P, Mennini T, Gelosa P, Tremoli E (2007). The interleukin-8 (IL-8/CXCL8) receptor inhibitor reparixin improves neurological deficits and reduces long-term inflammation in permanent and transient cerebral ischemia in rats. Mol Med.

[266] Shohami E, Gallily R, Mechoulam R, Bass R, Ben-Hur T (1997). Cytokine production in the brain following closed head injury: dexanabinol (HU-211) is a novel TNF-α inhibitor and an effective neuroprotectant. J Neuroimmunol.

[267] Tobinick E, Kim NM, Reyzin G, Rodriguez-Romanacce H, DePuy V (2012). Selective TNF inhibition for chronic stroke and traumatic brain injury. CNS drugs.

[268] Spera PA, Ellison JA, Feuerstein GZ, Barone FC (1998). IL-10 reduces rat brain injury following focal stroke. Neurosci Lett.

[269] Sewell DL, Nacewicz B, Liu F, Macvilay S, Erdei A, Lambris JD, Sandor M, Fabry Z (2004). Complement C3 and C5 play critical roles in traumatic brain cryoinjury: blocking effects on neutrophil extravasation by C5a receptor antagonist. J Neuroimmunol.

[270] Leinhase I, Schmidt OI, Thurman JM, Hossini AM, Rozanski M, Taha ME, Scheffler A, John T, Smith WR, Holers VM (2006). Pharmacological complement inhibition at the C3 convertase level promotes neuronal survival, neuroprotective intracerebral gene expression, and neurological outcome after traumatic brain injury. Exp Neurol.

[271] Brait VH, Rivera J, Broughton BR, Lee S, Drummond GR, Sobey CG (2011). Chemokine-related gene expression in the brain following ischemic stroke: no role for CXCR2 in outcome. Brain Res.

[272] Gross CE, Bednar MM, Howard DB, Sporn MB (1993). Transforming growth factor-beta 1 reduces infarct size after experimental cerebral ischemia in a rabbit model. Stroke.

[273] CLARK RS, CARLOS TM, SCHIDING JK, BREE M, FIREMAN LA, DeKOSKY ST, KOCHANEK PM (1996). Antibodies against Mac-1 attenuate neutrophil accumulation after traumatic brain injury in rats. J Neurotrauma.

[274] Parmentier S, Böhme GA, Lerouet D, Damour D, Stutzmann JM, Margaill I, Plotkine M (1999). Selective inhibition of inducible nitric oxide synthase prevents ischaemic brain injury. Br J Pharmacol.

